# A Series of Robust Copper-Based Triazolyl Isophthalate MOFs: Impact of Linker Functionalization on Gas Sorption and Catalytic Activity †

**DOI:** 10.3390/ma10040338

**Published:** 2017-03-24

**Authors:** Ulrike Junghans, Merten Kobalz, Oliver Erhart, Hannes Preißler, Jörg Lincke, Jens Möllmer, Harald Krautscheid, Roger Gläser

**Affiliations:** 1 Institute of Chemical Technology, Universität Leipzig, Linnéstr. 3, 04103 Leipzig, Germany; ulrike.junghans@uni-leipzig.de; 2 Institute of Inorganic Chemistry, Universität Leipzig, Johannisallee 29, 04103 Leipzig, Germany; merten.kobalz@chemie.uni-leipzig.de (M.K.); O.Erhart@web.de (O.E.); joerglincke@gmx.de (J.L.); krautscheid@rz.uni-leipzig.de (H.K.); 3 Institut für Nichtklassische Chemie e.V., Permoserstr. 15, 04318 Leipzig, Germany; preissler@inc.uni-leipzig.de (H.P.); moellmer@inc.uni-leipzig.de (J.M.)

**Keywords:** triazolyl isophthalate MOFs, crystal structures, structure-property relationship, linker substitution pattern, cyclohexene oxidation, heterogeneous catalysis

## Abstract

The synthesis and characterization of an isomorphous series of copper-containing microporous metal-organic frameworks (MOFs) based on triazolyl isophthalate linkers with the general formula ∞3[Cu_4_(*μ*_3_-OH)_2_(R^1^-R^2^-trz-ia)_3_(H_2_O)*_x_*] are presented. Through size adjustment of the alkyl substituents R^1^ and/or R^2^ at the linker, the impact of linker functionalization on structure-property relationships was studied. Due to the arrangement of the substituents towards the cavities, the porosity (pore fraction 28%–39%), as well as the pore size can be adjusted by the size of the substituents of the triazole ring. Thermal analysis and temperature-dependent PXRD studies reveal a thermal stability of the MOFs up to 230 °C due to increasing framework stability through fine-tuning of the linker substitution pattern. Adsorption of CO_2_ (298 K) shows a decreasing maximum loading with increasing steric demand of the substituents of the triazole ring. Furthermore, the selective oxidation of cyclohexene with *tert*-butyl hydroperoxide (TBHP) is studied over the MOFs at 323 K in liquid chloroform. The catalytic activity increases with the steric demand of the substituents. Additionally, these isomorphous MOFs exhibit considerable robustness under oxidizing conditions confirmed by CO_2_ adsorption studies, as well as by the catalytic selective oxidation experiments.

## 1. Introduction

Since the 1990s, metal-organic frameworks (MOFs) have emerged as an attractive class of porous materials for a broad spectrum of applications, like gas storage [1,2,3,4] and separation [5,6,7,8,9], as well as sensor design [10], biomedicine [11] and heterogeneous catalysis [12,13,14,15]. This versatile applicability derives from their large specific surface area, tunable properties, as well as their structural and chemical diversity [16,17]. However, in contrast to conventional materials, such as zeolites, the major drawback of MOFs is their limited thermal, hydrothermal and chemical stability [12]. Hence, in recent years, increasing attention has been paid to systematic investigations on isostructural MOFs by linker functionalization [18,19,20,21], metal ion substitution [22] and post-synthetic modification [23,24,25,26]. Typically, these modified MOFs show improved material properties, such as higher selectivity in terms of gas separation [27,28] or higher catalytic activity [29]. Furthermore, the hydrothermal stability of MOFs can be improved through linker functionalization with alkyl groups, resulting in an increased hydrophobic nature of the MOF surface [13,30,31]. Further, stability towards water, as well as oxidizing conditions is often required for the application of the MOFs as catalysts in the selective oxidation of organic substrates. For instance, several MOFs with nitrogen-containing aromatic moieties within the linker exhibit considerable robustness under these conditions [32,33].

Among the MOF-based catalysts for selective oxidations, those containing copper as the active element bear particular potential. Thus, the well-known Cu_3_(BTC)_2_ (BTC: 1,3,5-benzenetricarboxylate; also known as HKUST-1, HKUST: Hong Kong University of Science and Technology) was extensively studied, e.g., in the oxidation of xanthene [34], benzylic alcohols [35] or cyclooctene [36]. The “Cu-MOFs” [Cu(bpy)(H_2_O)(bpy)(BF_4_)_2_] [37], [Cu_2_(bipy)_2_(btec)] [38], [Cu(bipy)(H_2_btec)] [39,40], phenoxyacetic acid derivatives [Cu(L_1_)_2_(H_2_O)_2_], [Cu(L_1_)_2_(H_2_O)(Py)_2_], [Cu(L_3_)(H_2_O)Cl] [41], [Cu_2_(OH)(BTC)(H_2_O)]*_n_*·2*n*H_2_O [42], {[Cu_3_Ln_2_-(oda)_6_(H_2_O)_6_]·*n*H_2_O}*_n_* [43] and {[Cu_0.5_La_2_(HPDC)(PDC)_2_-(SO_4_)(H_2_O)_2_]H_2_O}*_n_* [44] also show catalytic activity for the selective oxidation of linear or cyclic alkenes with molecular oxygen or *tert*-butyl hydroperoxide (TBHP).

However, systematic studies on the adsorption and catalytic properties of MOFs by only one type of functionalization in the organic linker are still rare. The present work was, thus, devoted to the synthesis and investigation of the crystal structure, the thermal and adsorptive properties, as well as the catalytic activity of a series of copper-containing isomorphous MOFs based on triazolyl isophthalate linkers [45,46,47,48,49] with the general formula ∞3[Cu_4_(*μ*_3_-OH)_2_(R^1^-R^2^-trz-ia)_3_(H_2_O)*_x_*]. The ligands differ in their steric demand of the triazole substituents R^1^ and R^2^, i.e., R = H, Me, Et. This allows a comprehensive investigation of the impact of gradual changes in the linker on the MOF structure and porosity, as well as on gas adsorption, thermal and catalytic properties. The influence of different functional groups within the triazolyl isophthalate linker on the gas sorption properties was already observed recently with a related series of MOFs based on the paddle wheel motif [48]. In addition, triazole-based MOFs were shown to be active catalysts in the selective oxidation of cyclooctene and cyclohexene with TBHP [36,50,51]. Accordingly, the selective oxidation of cyclohexene with TBHP in the liquid phase was chosen as a test reaction in the present work to investigate the influence of the triazole substituents on the catalytic activity of the MOFs. In addition, this conversion is a widely-studied test reaction to investigate the catalytic activity of newly-developed oxidation catalysts, since the formation of the oxidation products strongly depends on the chosen reaction conditions, as well as the used catalyst [52,53,54]. Thus, a direct comparison of the obtained results to already published ones is possible. Lastly, the catalytic activity, selectivity and stability of ∞3[Cu_4_(*μ*_3_-OH)_2_(R^1^-R^2^-trz-ia)_3_(H_2_O)*_x_*] catalysts is directly compared to that of the reference materials Cu_3_(BTC)_2_ and Cu(NO_3_)_2_.

## 2. Results and Discussion

### 2.1. Synthesis and X-ray Crystallography of ***1**–**5***

Based on five triazolyl isophthalate ligands [47,48] with methyl and ethyl substituents, including mono- and di-substituted linkers, fine-tuning of the steric demand of the ∞3[Cu_4_(*μ*_3_-OH)_2_(R^1^-R^2^-trz-ia)_3_(H_2_O)_x_] MOFs (**1**–**5**) is possible (Table 1). The crystal structures of **3**–**5** were determined by single crystal X-ray diffraction. Suitable crystals were obtained starting from the protonated ligands and Cu(OAc)_2_ or CuSO_4_ via solvothermal synthesis using H_2_O, H_2_O/MeCN or H_2_O/MeOH as solvents. In addition, compounds **1**–**5** are easily accessible in the gram scale by refluxing a solution of the starting materials in H_2_O/MeOH, in the case of **4** even in the “green” solvent H_2_O. **3**–**5** crystallize in the orthorhombic space group *Pbca* (No. 61) with eight formula units (Z) per unit cell (Table 2). According to the X-ray powder diffraction (PXRD) patterns, **1** and **2** complete the series of isomorphous MOFs **1**–**5**, but are not accessible as single crystals. Detailed MOF synthesis protocols and crystal structure data are reported in the Electronic Supporting Information (ESI, Tables S1 and S2).

**Figure 1:**
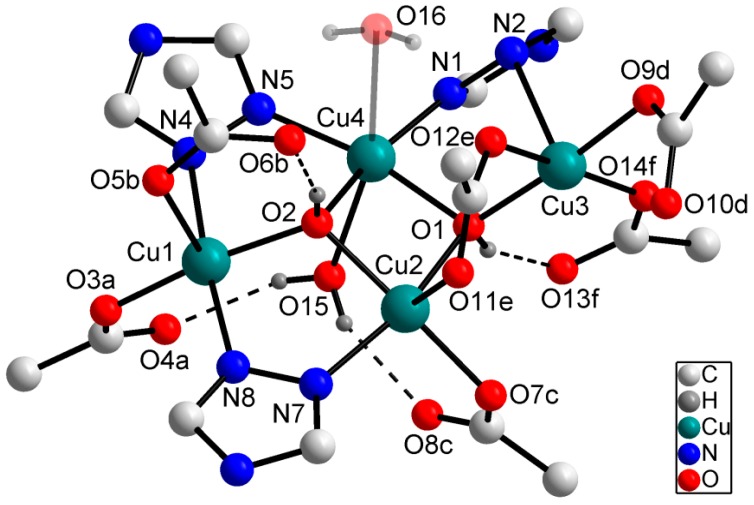
Structural motif of **3**–**5**: Coordination environment of the Cu^2+^ ions. The substituents of the triazole rings are omitted for clarity. The coordinating water molecule O16 is present only in the crystal structure of **4**. Symmetry codes: a: x, 1.5 − y, −0.5 + z; b: −0.5 + x, y, 1.5 − z; c: 1.5 − x, −0.5 + y, z; d: 1 − x, 1 − y, 1 − z; f: 1.5 − x, 1 − y, 0.5 + z.

The asymmetric unit of **3**–**5** contains three linker molecules, one [Cu_4_(*μ*_3_-OH)_2_]^6+^ unit and one (**3**, **5**) or two (**4**) coordinating water molecules. The secondary building unit (SBU) is formed by four Cu^2+^ ions and two triple bridging hydroxide ions (Figure 1). Additionally, the Cu^2+^ ions are bridged by three triazole groups and one carboxylate group (O11e, O12e). The four Cu^2+^ ions in the crystal structures of **3** and **5** show a distorted square pyramidal coordination sphere. Due to coordination of a second water molecule in the case of **4**, the Cu^2+^ ion Cu4 shows a distorted octahedral coordination environment (cf. (confer) Figure 1). All of these polyhedra are elongated, as expected for the d^9^ electron configuration according to the Jahn-Teller theorem. This is illustrated by the significantly longer bonds in apical positions compared to bond lengths in equatorial positions (Table 3). The open metal sites of the Cu^2+^ ions are shielded by triazole substituents and the steric demand of the carboxylate groups. As shown in Figure 1, hydrogen bonds are built up by coordinating water molecules, bridging hydroxide ions and carboxylate groups.

Each [Cu_4_(*μ*_3_-OH)_2_]^6+^ SBU is coordinated by nine triply bridging linker molecules (three triazole groups, six carboxylate groups). Topological analysis of the three-dimensional network reveals the point symbol {4.6^2^}_2_{4^2^.6}{4^7^.6^22^.8^7^}, which is not assigned to a known topology [55].

**Figure 2:**
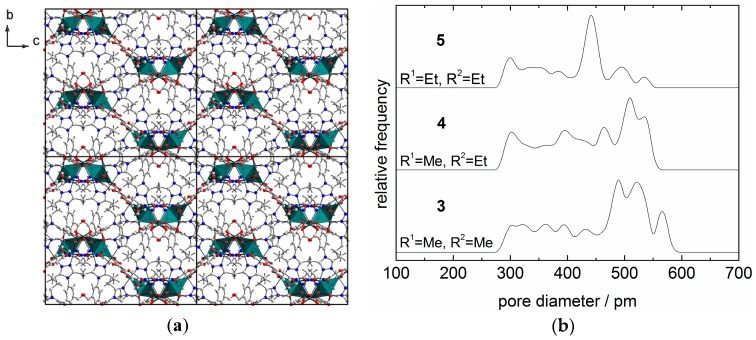
Arrangement of [Cu_4_(*μ*_3_-OH)_2_]^6+^ units in the three-dimensional network of **4** (2·2·2 supercell, viewed along the crystallographic *a*-direction (**a**); and comparison of the calculated pore size distributions (PSDs, [57,58,59]) of **3**–**5** (**b**).

The pore fraction of **3**–**5** calculated from the crystal structure data is determined as 39% (**3**), 34% (**4**) and 28% (**5**), respectively [56]. As a representative example, the 3D packing in the crystal structure of **4** is shown in Figure 2. This packing diagram and also diagrams with other viewing directions (not shown) show that there are no straight pore channels in these crystal structures. Consequently, the pores are not easily visible by looking at these diagrams. More information can be gained from the calculations of pore size distributions (PSDs, Figure 2). The PSDs of **3**–**5** calculated on the basis of the crystal structure data show a multimodal shape with pore sizes in the range of 300–580 pm (Figure 2) [57,58,59]. This confirms that pores are present within the investigated MOFs. Hence, **3**–**5** represent microporous materials. Due to the arrangement of the triazole substituents towards the cavities (Figure 2), the maximum pore size can be fine-tuned by the choice of the substituent. Therefore, targeted modification of the pore system is possible by adjusting the substitution pattern of the triazole ring.

### 2.2. X-ray Powder Diffraction and Thermal Stability of ***1**–**5***

**Figure 3:**
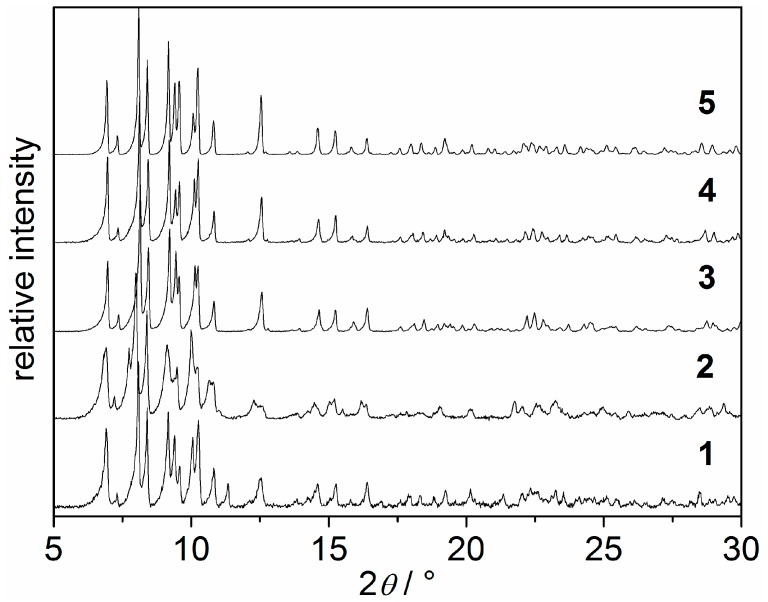
X-ray powder diffraction patterns (*λ*(Cu-K_α1_) = 154.060 pm) of **1**–**5**.

As mentioned above, compounds **1** and **2** are not accessible as single crystals. Hence, single crystal X-ray analyses of these compounds could not be performed. However, as presented in Figure 3, Figures S1 and S4, the powder X-ray diffraction patterns of **1** and **2** closely resemble those of compounds **3**–**5**. Based on these data, **1**–**5** can be regarded as isomorphous. Due to the overlap of the broadened reflections, indexing of the PXRD patterns of **1** and **2** was not possible. However, as the PXRD patterns of **1**–**5** are in good agreement except for minor deviations of intensity proportions of individual reflections (Figure 3), it can be concluded that both mono- and disubstituted triazolyl ligands are tolerated by the network.

The PXRD patterns of **4** and **5** after various post-synthetic treatments (Soxhlet extraction with methanol, adsorption experiments, adsorption experiment and resolvation with methanol, catalytic test, catalytic test and resolvation with methanol, evacuation or after 16 h in boiling water) illustrate the robustness of these materials (Figure S2; comprehensive PXRD data of **1**–**3** are shown in Figure S1). As a representative example, **5** was chosen for the investigation of the stability of the framework materials towards hot liquid water, as this is a key requirement for the application of MOFs in adsorption processes and as heterogeneous catalysts. Although the synthesis of **5** requires the presence of methanol as the organic solvent, the PXRD pattern remains unchanged even after 16 h in boiling water. Further, the PXRD pattern of **4** shows no deviation from that of the pristine material pattern even in the evacuated state after activation under vacuum at 313 K for 24 h. Due to this remarkable robustness, it can be concluded that **1**–**5** are rigid frameworks of the second generation according to the classification of Kitagawa et al. [16,60,61].

The thermal stability of the microporous framework materials **1**–**5** after Soxhlet extraction with methanol was studied by temperature-dependent X-ray powder diffraction (TD-PXRD) and simultaneous thermal analysis (TG-DTA-MS). The reflection positions of **1**, **2** and **5** remain unchanged with increasing temperature (Figure S4). In contrast, the TD-PXRD patterns of **3** and **4** show slight changes of individual reflection positions at elevated temperatures. However, a distinct phase change is not observed for **1**–**5**, illustrating the robustness of the framework structures. The TD-PXRD patterns reveal a loss of crystallinity in the temperature range between 180 °C (**2**) and 230 °C (**5**).

**Figure 4:**
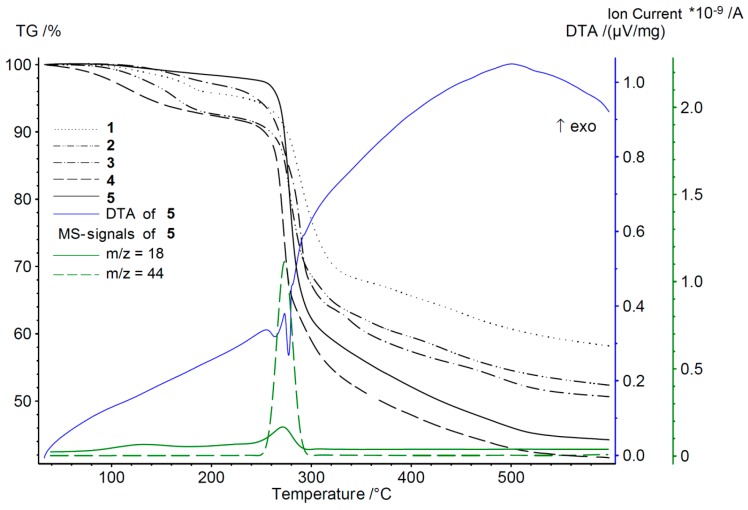
Simultaneous thermal analyses (TG-DTA-MS) of **1**–**5** after Soxhlet extraction with methanol. MS signals (**5**) of (H_2_O)^+^ (*m*/*z* = 18) and (CO_2_)^+^ (*m*/*z* = 44) illustrate the evaporation of guest molecules and the decomposition of the framework, respectively.

A comparative illustration of the TG-DTA-MS analyses of **1**–**5** after Soxhlet extraction with methanol is shown in Figure 4 (for individual diagrams and a more detailed description, see ESI 2.2 and Figures S5–S7). All materials show a mass loss up to 200 °C, which is related to the evaporation of guest molecules. Although all materials were Soxhlet extracted with methanol, the evaporation of water is still detected by MS at elevated temperatures. The multi-stepped water loss results from different degrees of adsorptive interaction with the walls of the pores of various sizes. The last peak in the MS signal of water (*m*/*z* = 18 (H_2_O)^+^) is assigned to the coordinated water molecules and bridging hydroxide ions. This water loss is accompanied by the thermal decomposition of the frameworks, associated with the release of CO_2_ from the organic linker.

The decomposition temperatures determined by TG-DTA-MS analysis are in good agreement with the values found by TD-PXRD (Figure S8). With increasing steric demand of the triazole substituents, a slight increase of the decomposition temperature from **1** (220 °C; R^1^ = H, R^2^ = Me) to **5** (250 °C; R^1^ = R^2^ = Et) is observed. This is most likely due to the reduced porosity and pore size and, thus, a reduced fragility of the framework.

### 2.3. Adsorption of CO_2_

**Figure 5:**
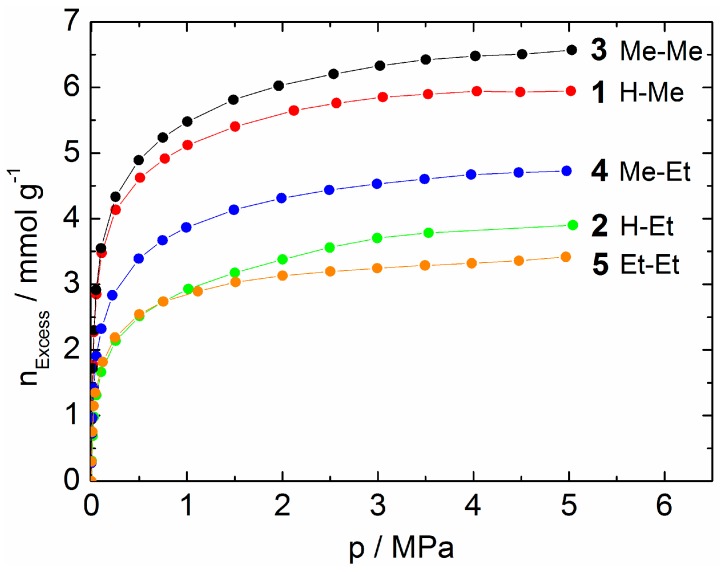
CO_2_ adsorption isotherms (298 K) of **1**–**5** (lines are to guide the eyes).

In order to evaluate the adsorption characteristics of **1**–**5**, high-pressure CO_2_ (298 K) and low-pressure N_2_ (77 K) adsorption studies were performed. The CO_2_ adsorption isotherms of **1**–**5** correspond to type I of the IUPAC classification of physisorption isotherms (Figure 5 and Figure S9) [62,63]. Obviously, the choice of the substituent clearly influences the saturation loading of CO_2_. However, the expected decrease of the maximum loading with increasing steric demand of the substituent on the triazole ring is not apparent at first sight. By detailed analysis of the pore volumes, this deviation can be explained.

As expected, the experimentally determined specific pore volume of **1** (R^1^ = H, R^2^ = Me, 0.33 cm^3^ g^−1^) is slightly larger compared to the calculated value of **3** (R^1^ = R^2^ = Me, 0.31 cm^3^ g^−1^). Based on the specific pore volumes of **3**–**5** calculated from the crystal structure data, an increment of approximately 0.05 cm^3^ g^−1^ for a CH_2_ extension of the alkyl substituent is estimated (Table 4). Thus, the calculated pore volume of **1** is estimated to be approximately 0.36 cm^3^ g^−1^, which is somewhat larger compared to the experimental value. The experimentally determined specific pore volume of **2** (0.22 cm^3^ g^−1^) is significantly smaller compared to that of **1** and even smaller than the value found for **4** (R^1^ = Me, R^2^ = Et, 0.26 cm^3^ g^−1^). It is, however, in quite good agreement with the specific pore volume of **5** (R^1^ = R^2^ = Et). This finding gives rise to the assumption that the ethyl group in the structure of **2** is located towards the cavities and causes partial blocking of the pore system. Introduction of a second ethyl group (**5**) causes no significant pore volume reduction. This is in good agreement with the pore size distributions calculated based on single crystal structure data (Figure 2). A CH_2_ extension of the methyl group (from **3** to **4**) leads to a reduction of the maximum pore size. In contrast, the extension of the second methyl group (from **4** to **5**) does not cause an additional contraction of the pore. In the case of **4**, the calculated pore volume is confirmed experimentally (0.26 cm^3^ g^−1^), while for **5**, a slightly smaller value is found (Table 4).

A peculiarity is found for **3**. The experimentally-determined specific pore volume of this material (0.36 cm^3^ g^−1^) is significantly larger than the calculated value based on crystal structure data (0.31 cm^3^ g^−1^). A possible explanation for this result is a desolvated phase with a larger pore fraction compared to the structure of the as-synthesized material. However, a structural change during CO_2_ adsorption is unlikely, as this process would cause a stepwise uptake.

For the isomorphous MOF series **1**–**5**, the CO_2_ adsorption capacity can be modified by fine-tuning of the substitution pattern of the triazole ring. The expected decrease of the maximum loading with increasing steric demand of the substituent is experimentally confirmed for **1** (R^1^ = H, R^2^ = Me), **4** (R^1^ = Me, R^2^ = Et) and **5** (R^1^ = R^2^ = Et). The low pressure N_2_ adsorption isotherms (77 K) correspond to type I of the IUPAC recommendations, as well. They show a steep increase of adsorbed volume followed by a pronounced plateau (Figure S10). However, in contrast to CO_2_, N_2_ is not a proper probe molecule to experimentally prove the different specific pore volumes of **1**–**5**.

### 2.4. Catalytic Selective Oxidation of Cyclohexene with TBHP over ***1**–**5***

**Figure 6:**
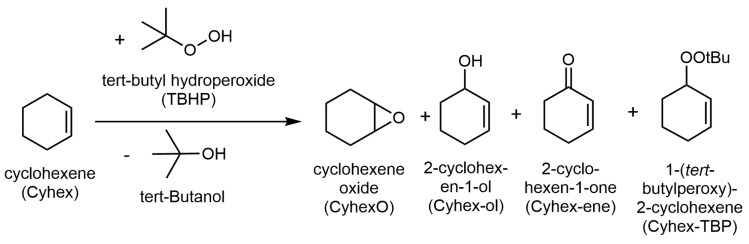
Reaction scheme of catalytic oxidation of cyclohexene with *tert*-butyl hydroperoxide (TBHP).

**Figure 7:**
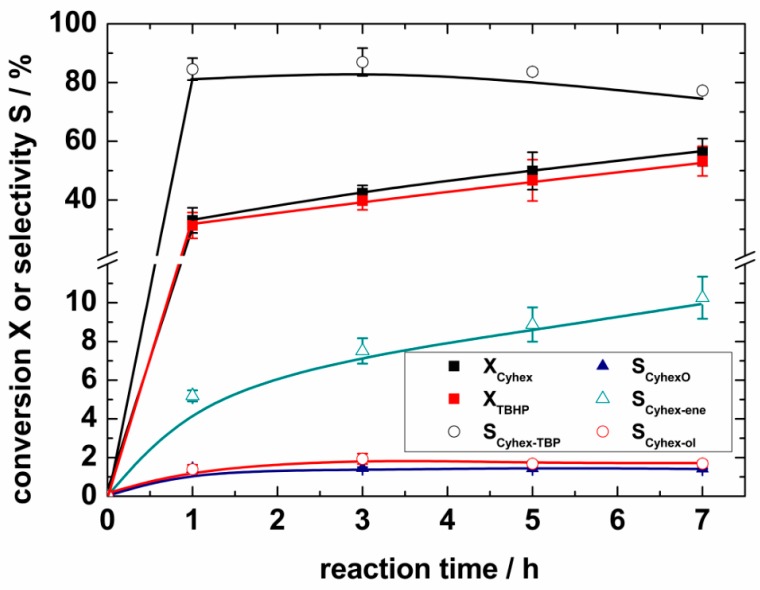
Conversion of cyclohexene X_Cyhex_ and TBHP X_TBHP_, as well as selectivity for 1-(*tert*-butylperoxy)-2-cyclohexene S_Cyhex-TBP_, cyclohexene oxide S_CyhexO_, 2-cyclohexen-1-one S_Cyhex-ene_, 2-cyclohexen-1-ol S_Cyhex-ol_ for **5** as a function of reaction time in liquid chloroform (reaction conditions as in Table 5).

The MOFs **1**–**5** were studied as catalysts for the conversion of cyclohexene (Cyhex) with TBHP in liquid chloroform (Figure 6). As an example, Figure 7 shows the conversion of Cyhex and TBHP, as well as the selectivity for the corresponding reaction products over **5** in dependence of the reaction time. After 7 h, 56% Cyhex and 53% TBHP are converted. Concomitantly, the selectivity for Cyhex-TBP decreases from 85% after 3 h of reaction to 77% after 7 h suggesting further conversion to 2-cyclohexen-1-one, the selectivity of which reaches 10%. Other byproducts, cyclohexene oxide and 2-cyclohexen-1-ol, are formed in negligible amounts only (selectivity of 1% and 2%, respectively). The observed product distribution suggests a similar reaction mechanism to that reported by Tonigold et al. [66], who investigated the selective cyclohexene oxidation under solvent-free conditions over the Co-containing MOF MFU-1 (MFU: Metal-organic framework Ulm University). They explained the formation of the reaction products via a radical-dominated reaction pathway involving the formation of *tert*-butoxyl and *tert*-butylperoxyl radicals at the metal sites of the MOF.

The remaining 10% of the product selectivity is assumed to correspond to the formation of 3-methylcyclohexene (3%), which is formed through a side reaction of Cyhex with the solvent chloroform and the formation of higher molecular weight products (1%), such as polyperoxides or oligomers of Cyhex, which cannot be detected via GC analysis. In addition, the strong adsorption of the oxygenated products at the Cu sites [67,68,69] adds to the remaining product selectivity (3%). The still “missing” 3% selectivity is within the experimental accuracy of the catalytic experiments. A similar product distribution was also observed for the other Cu-containing MOFs **1**–**4**, as well as for the reference catalysts Cu_3_(BTC)_2_ and Cu(NO_3_)_2_ (conversion of Cyhex and TBHP and selectivity for Cyhex-TBP, CyhexO, Cyhex-ene, Cyhex-ol for **1**–**5**, as well as Cu(NO_3_)_2_ and Cu_3_(BTC)_2_ are shown in ESI, Figure S11). However, in contrast to **5**, the selectivities for Cyhex-TBP found for **1**–**4** and Cu_3_(BTC)_2_ are nearly the same and essentially independent of the cyclohexene conversion (Figure S12). Hence, assuming that the same reaction mechanism is valid for all the investigated solid catalysts, **5** probably exhibits a higher activity. This is confirmed by comparison of the TOFs obtained over **1**–**5** summarized in Table 5. While a similar activity (within experimental accuracy) over **1**–**4** compared to Cu_3_(BTC)_2_ is observed, an up to 1.5-fold higher activity is reached in the presence of **5**. Interestingly, the highest catalytic activity is observed for the MOF with the highest steric demand of the triazole substituents. However, among the studied catalysts, **5** has the lowest specific pore volume and specific surface area, as well as a relatively narrow maximum pore size of 540 pm (cf. Table 4 and Figure 2; Cu_3_(BTC)_2_: pore size = 1.6/1.1, 0.6 nm [70,71]; V_micro_ = 0.62 cm^3^ g^−1^, S_BET_ = 1526 m^2^ g^−1^). Hence, no clear connection between the textural properties of the studied triazolyl-based MOFs and the catalytic activity becomes obvious at first sight. In fact, different aspects probably contribute to the higher activity of **5**. According to the kinetic diameter of cyclohexene (0.6 nm [72]), which exceeds the maximum pore size of **5** (540 pm) the reaction could take place at the outer surface of **5**. Nevertheless, **5** shows a similar activity to the Cu^2+^ ions from the dissolved Cu(NO_3_)_2_, and both catalysts were added with the same molar amount of Cu to the reaction solution; the selective oxidation of cyclohexene with TBHP likely takes place within the pores of **5**. In the case of **1**–**4**, the oxidation probably occurs mainly at the outer surface of the catalysts as they exhibit a lower activity than Cu(NO_3_)_2_, suggesting that only a part of the active sites are accessible for the reactants. Secondly, **5** has the most narrow maximum pore size (Figure 2), which could cause diffusion limitations within the pores, resulting in elongated contact times of the reactant on the active sites. In addition, while the specific surface area of **5** is approximately one half of the surface areas of **3** and **4**, no significant difference of the specific pore volume can be observed in **4** and **5** (Table 4). Hence, **5** exhibits a relatively higher surface area within the pores, also contributing to the higher catalytic activity. At last, due to the different triazole substituents, the intrinsic activity of the Cu sites is probably also different, resulting in different Lewis acidity and, thus, catalytic properties for **1**–**5**.

Overall, although linker functionalization with alkyl groups can significantly improve the hydrothermal stability and adsorption properties of the isomorphous series of MOFs, a fine-tuning of their catalytic activity merely based on the difference in steric demand is not evident.

Nevertheless, the catalytic activity of **1**–**5** is within the range of results for the cyclohexene conversion with TBHP and Cyhex-TBP as the main reaction product as reported in the literature (Table 6). However, it has to be noted that, in contrast to **1**–**4**, cyclohexene is able to access the pores of MFU-1 [73] and [Co^II^(BPD)]·3DMF [74] (Table 6) (pore width of 1.8 nm for MFU-1 [73] and 1.1 nm, as well as 4.5–7.0 nm respectively for [Co^II^(BPD)]·3DMF [74]). Hence, MFU-1 and [Co^II^(BPD)]·3DMF show a superior catalytic activity compared to **1**–**4** as the higher molar amount of active sites within **1**–**4** (0.46 mmol vs. 0.095 mmol/0.056 mmol respectively) is not fully accessible for all reactants. In addition, other Cu-containing MOFs show an overall higher selectivity for Cyhex-ene [42,44] and CyhexO [39,40] with TBHP or molecular oxygen as the oxidant. As can be further seen from Table 6, [Cu_2_L_2_] (L = bis(carboxyphenyl)-1,2,4-triazole) [50] even shows an up to 1.5-fold higher activity in comparison to **5** at the same reaction conditions, although both reactions could occur on the outer surface of the catalysts. This could be again caused by the different coordination environment of the catalytically active sites resulting in different intrinsic activity of the Cu sites and, thus, different Lewis acidity. In addition, low coordinated Cu sites at the outer surface of [Cu_2_L_2_] probably cause the higher activity compared to **1**–**5**, as these active sites are easily accessible. Furthermore, Kobalz et al. [50] concluded that the conversion of cyclohexene probably occurs at the outer surface of [Cu_2_L_2_]. The previous comparison of the catalytic results of **1**–**5** to already published results (cf. Table 6) emphasizes that for the development of highly active and selective MOF-based catalysts, not only the steric demand of the linker substituents, but also the resulting pore structure, as well as the design of the coordinative and electronic environment of the active sites need to be carefully considered.

The crystal structure remains intact for **2**, **4** and **5** during the catalytic experiment. In contrast to Cu_3_(BTC)_2_, no changes in reflection intensity or width are observed for the PXRD patterns of **2**, **4** and **5** (Figures S1 and S2). In the case of Cu_3_(BTC)_2_, the reflection intensity decreases probably due to remaining solvent molecules or adsorbed reactants or products from the conversion (Figure S3). However, a broadening of the reflections occurs for **1** and **3** (Figure S1). Even after resolvation with methanol, the PXRD patterns of **1** and **3** do not resemble the ones of the as-synthesized materials, indicating changes in the crystal structure during the catalytic conversion. These changes could also be a reason for the lowest catalytic activity of **1** and **3** within the investigated MOFs.

In another experiment, the heterogeneous nature of the catalytic conversion was proven by hot filtration of **5** and **3** (Figure S13). In contrast to **3**, for **5**, a further, but less pronounced progress of conversion was observed after filtration of the catalyst (after 3 h) until 5 h of reaction due to the action of radicals. These are generated on the catalyst surface during the first 3 h of the experiment. After 5 h of reaction, the conversion lies within the range of the reaction without any added catalyst (Figure S13). After hot filtration, no copper for the reaction over 5 and 0.2 wt % of the copper in **3** was detected via elemental analysis by optical emission spectrometry with inductively coupled plasma (ICP-OES). However, the small amount of 0.05 mg (0.2 wt %) Cu, which leached from **3**, was not able to further catalyze the conversion of cyclohexene with TBHP. Importantly, like Cu_3_(BTC)_2_, **3** and **5** could be regenerated by Soxhlet extraction. A second activation in vacuum at 323 K and reuse in another catalytic run revealed no loss of activity and selectivity within the experimental accuracy (Figure S14). Therefore, the pore system of **3** and **5** regenerated after the catalytic conversion is still accessible, as can be seen from the CO_2_ isotherms (Figure S15). The previously mentioned results confirm the stability of the investigated catalysts and the heterogeneous nature of the reaction.

## 3. Materials and Methods

### 3.1. Synthesis of ∞3[Cu_4_(μ_3_-OH)_2_(R^1^-R^2^-trz-ia)_3_(H_2_O)_x_] (R^1^ = H, Me, Et; R^2^ = Me, Et; x = 1, 2; ***1**–**5***)

All reagents and solvents were purchased from commercial sources and used without further purification to synthesize the protonated ligands, as well as metal-organic frameworks. The protonated ligands were synthesized analogously to published procedures [45,47,48]. For solvothermal synthesis of **3**, **4** and **5**, steel autoclaves with appropriate polytetrafluoroethene inserts were used. Additionally, **1**–**5** are accessible as microcrystalline powders by refluxing equimolar amounts of Cu(OAc)_2_·H_2_O (98%, Sigma-Aldrich, Taufkirchen, Germany) or CuSO_4_·5H_2_O (98%, Sigma-Aldrich) and the appropriate ligand in H_2_O or a H_2_O/MeOH (99.8%, AnalaR NORMAPUR^®^ ACS, VWR, Darmstadt, Germany) or H_2_O/MeCN (99.9%, CHROMASOLV^TM^, VWR, Darmstadt, Germany) mixture (1:1, *v*/*v*). Solvent exchange of the as-synthesized samples was carried out via subsequent Soxhlet extraction with methanol (99.8%, AnalaR NORMAPUR^®^ ACS, VWR, Darmstadt, Germany). Detailed synthesis procedures and analytical data (PXRD, TD-PXRD, TG-DTA-MS) are reported in the ESI.

Cu_3_(BTC)_2_ purchased from Sigma-Aldrich and activated at 393 K for 16 h in ambient air, as well as the Cu^2+^ salt Cu(NO_3_)_2_ (99.5%, Merck, Darmstadt, Germany) were included in the catalytic experiments.

### 3.2. Characterization of ***1**–**5***

Single crystals of **3**, **4** and **5** were fixed with Fomblin^®^ oil in the center of a plastic loop and mounted on a STOE IPDS-2T image plate diffractometer (Mo-K_α_ λ = 71.073 pm; Stoe & Cie GmbH, Darmstadt, Germany). The data sets were processed by STOE X-Area (Stoe & Cie GmbH, Darmstadt, Germany) [75]. Crystal structures were solved by direct methods and refined using SHELX-2014 [76]. Positions of the framework hydrogen atoms were calculated for geometrically idealized positions. Contributions from disordered solvent molecules were removed by the SQUEEZE routine of the program package PLATON (Utrecht University, Utrecht, Netherlands) [56]. For specification of disordered atoms, split-position models were used. Crystal structure data and the results of the structure refinements are summarized in the ESI (Table S2). The program DIAMOND 3.2f (Crystal Impact GbR, Brandenburg, Germany) was used to visualize the structures [77]. CCDC 1526752–1526754 contain the supplementary crystallographic data. These data can be obtained free of charge from the Cambridge Crystallographic Data Centre via www.ccdc.cam.ac.uk/data_request/cif.

Prior to each adsorption experiment, the Soxhlet-extracted samples were activated in vacuum at 323 K for 24 h. Nitrogen adsorption experiments were performed at 77 K with the commercially available volumetric adsorption analyzer BELSORP-max (MicrotracBEL Corp., Paris, France). High-pressure CO_2_ adsorption isotherms were recorded on a magnetic suspension balance (Rubotherm GmbH, Bochum, Germany) at 298 K. Various pressure transducers (Newport Electronics GmbH, Deckenpfronn, Germany) were used in the range of vacuum (*p* < 0.05 Pa) up to 10 MPa. Each adsorption isotherm was taken at least twice to ensure the reproducibility of the data (within ±5% of the reported values). The ESI (cf. Section 2.3) contains a detailed description of these experiments.

Detailed description of PXRD measurements [78], TG-DTA-MS analyses, as well as elemental analyses of **1**–**5** are reported in the ESI (cf. Section 2.2 and Section 3.2).

### 3.3. Catalytic Selective Oxidation of Cyclohexene

The catalytic experiments were carried out as already described in a previous report [50]. Fifteen centimeters cubed of chloroform (99.8+%, Alfa Aesar, Karlsruhe, Germany) were loaded into a batch reactor followed by the addition of cyclohexene (2.054 g, 25 mmol, 99%, Sigma-Aldrich, Taufkirchen, Germany), *tert*-butyl-hydroperoxide (TBHP, 7.545 g, 50 mmol, 5.5 M solution in *n*-decane, Sigma-Aldrich) and chlorobenzene (2.814 g, 25 mmol, 99+%, Acros organics, Nidderau, Germany) as the internal analytical standard corresponding to a molar ratio of cyclohexene:TBHP:chloroform of 1:2:7. To start the reaction, the catalysts **1**–**5** (activated at 323 K for 16 h under vacuum) or the reference catalyst Cu_3_(BTC)_2_ (activated at 393 K for 16 h in ambient air) used in different quantities (Cu content of 0.46 mmol) were added to the reaction solution.

For recycling experiments, the catalyst was removed from the reaction mixture by centrifugation after the first run, Soxhlet extracted with methanol (99.8%, Merck, Darmstadt, Germany), reactivated as described above and, again, added to a fresh reactant solution. To investigate if leaching of the catalytic species occurs, the catalyst was removed from the reaction mixture by centrifugation at reaction temperature after 3 h (hot filtration). The remaining reactant solution was again subjected to the reaction conditions for four more hours.

Liquid samples (0.3 cm^3^) were taken from the reaction mixture at different time intervals. Aliquots of the samples (0.1 cm^3^) were diluted in 0.5 cm^3^ chloroform and analyzed by capillary gas chromatography (Shimadzu GC 2010 equipped with a flame ionization detector, Shimadzu, Duisburg, Gemany) using nitrogen as the carrier gas. Product separation was achieved on a capillary column (95% dimethylpolysiloxane cross-linked with 5% diphenylpolysiloxane, Restek RTX-5, 30-m length, 0.25-mm inner diameter, 0.25-μm film thickness). Reaction products were identified by co-injection of authentic samples and by GC-MS (GC Varian 3800, Agilent Technologies, Waldbronn, Germany). Data on the conversion of cyclohexene were reproducible within ±5%, of TBHP conversion within ±5%, selectivity for 1-(*tert*-butylperoxy)-2-cyclohexene within ±6% and selectivity for 2-cyclohexen-1-one ±1% (overall for 3 and 5). The turnover frequency TOF (in h^−1^) was calculated as the ratio of the amount of converted cyclohexene and of the amount of copper sites present in the catalyst and the reaction time in hours within a reproducibility of ±0.4 h^−1^.

## 4. Conclusions

The triazolyl isophthalate MOFs **1**–**5** represent a series of isomorphous MOFs; **3**–**5** were obtained as single crystals. Due to the arrangement of the triazole substituents towards the cavities, both the porosity and the pore size can be adjusted by the choice of the alkyl substituent. **3**–**5** are microporous materials with pore sizes in the range of 300–580 pm and specific pore volumes of 0.19–0.36 cm^3^ g^−1^. Similar specific pore volumes to the calculated ones were determined from CO_2_ adsorption isotherms. In contrast, N_2_ is not a suitable probe molecule to prove the impact of the steric demand of the triazole substituents on the accessible pore volume. **1**–**5** exhibit a thermal stability up to 230 °C. The framework stability is increased by a higher steric demand of the substituents. Additionally, **5** remains stable even after 16 h in boiling water.

A direct relation between the steric demand of the triazole substituents and the catalytic activity of **1**–**5** in the liquid phase selective oxidation of cyclohexene with TBHP is not apparent. Nevertheless, all five MOFs are catalytically active. **5** is the most active catalyst. It even shows a 1.5-fold higher activity compared to the reference catalyst Cu_3_(BTC)_2_ although its triazole substituent has the highest steric demand. Although, the cyclohexene conversion could partly take place at the outer surface of **5**, the pores appear to be accessible for the reactants. Moreover, the Cu sites in **5** are probably more active towards the promotion of a radical-based oxidation reaction than the Cu sites in Cu_3_(BTC)_2_. The comparable activity of **5** to that of the dissolved Cu(NO_3_)_2_ was rationalized by the cyclohexene conversion taking place within the micropores of **5**. Additionally, reusability and hot filtration measurements of **3** and **5** confirmed the stability of the investigated materials during oxidation catalysis. The crystal structure remains intact, and no significant pore blocking was observed after catalysis by CO_2_ adsorption analysis.

The systematic investigations of this study thus confirm that small changes within the MOF structure can cause distinct differences in their thermal, adsorptive and catalytic properties. Hence, the design of adsorbents with specific gas adsorption properties is possible. Furthermore, the high potential of Cu-containing MOFs for the selective oxidation of organic substrates due to fine-tuning of the catalytic properties is illustrated. Besides the steric demands, the design of the coordinative and electronic environment of the active Cu sites within MOF-based catalysts needs to be carefully considered.

## Figures and Tables

**Table 1:** Linkers with different substitution patterns in **1**–**5**.

**MOF**	**Ligand**	**R^1^**	**R^2^**	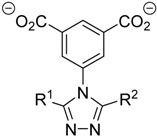
**1**	(H-Me-trz-ia)^2−^	H	Me	
**2**	(H-Et-trz-ia)^2−^	H	Et	
**3**	(Me_2_-trz-ia)^2−^	Me	Me	
**4**	(Me-Et-trz-ia)^2−^	Me	Et	
**5**	(Et_2_-trz-ia)^2−^	Et	Et	

**Table 2:** Space group, unit cell parameters (a, b, c), volume (V) and formula units (Z) per unit cell of **3**–**5**.

**MOF**	**3** (R^1^ = R^2^ = Me)	**4** (R^1^ = Me, R^2^ = Et)	**5** (R^1^ = R^2^ = Et)
space group	Pbca (No. 61)	Pbca (No. 61)	Pbca (No. 61)
a/pm	1875.00(8)	1872.71(3)	1854.69(5)
b/pm	2428.78(6)	2461.21(5)	2420.12(7)
c/pm	2540.75(7)	2525.07(6)	2547.32(7)
V/10^6^ pm^3^	11,570.6(7)	11,638.4(4)	11,433.8(6)
Z	8	8	8

**Table 3:** Selected distances and angles in the crystal structures of **3**–**5**.

MOF	3	4	5	MOF	3	4	5
	bond length/pm				angle/°		
Cu1-O2	193.2(4)	192.3(2)	192.7(2)	Cu3-O1	196.8(3)	194.7(3)	195.5(2)
Cu1-N4	202.1(4)	202.0(3)	201.5(2)	Cu3-O12e	195.6(4)	195.8(3)	195.1(2)
Cu1-O3a	194.1(4)	192.7(3)	194.7(2)	Cu3-O9d	196.4(4)	194.7(3)	194.3(2)
Cu1-N8	200.3(4)	202.6(3)	201.8(2)	Cu3-O14f	197.5(4)	196.1(3)	196.6(2)
Cu1⋯O5b	243.2(4)	256.6(4)	240.2(3)	Cu3⋯N2	246.9(5)	268.3(5)	256.2(3)
Cu2-O1	196.9(3)	195.9(2)	198.3(2)	Cu4-O1	197.6(3)	197.0(3)	198.0(2)
Cu2-O2	198.0(3)	197.1(3)	196.2(2)	Cu4-O2	196.8(4)	196.3(2)	195.7(2)
Cu2-O7c	195.0(4)	197.4(3)	195.1(2)	Cu4-N1	198.4(5)	198.5(3)	198.8(3)
Cu2-N7	201.4(5)	199.0(3)	201.8(3)	Cu4-N5	203.6(4)	201.0(3)	202.5(2)
Cu2⋯O11e	224.4(4)	221.9(3)	219.6(3)	Cu4⋯O15	230.0(4)	243.0(4)	225.4(3)
				Cu4⋯O16	-	266.0(5)	-
O2-Cu1⋯O5b	91.6(1)	84.9(1)	91.89(8)	O1-Cu3⋯N2	82.8(2)	79.7(1)	81.24(9)
O2-Cu1-N4	87.5(2)	88.1(1)	86.77(9)	O1-Cu3-O14f	95.7(2)	95.1(1)	96.01(9)
O2-Cu2⋯O11e	97.3(1)	99.0(1)	98.08(8)	O1-Cu4⋯O15	88.5(1)	86.8(1)	90.22(9)
O2-Cu2-O1	80.7(1)	81.2(1)	81.08(8)	O1-Cu4-O2	80.8(1)	81.1(1)	81.28(8)

**Table 4:** Calculated (cal.) and experimentally determined specific pore volumes of **1**–**5** (Gurvich equation, saturation vapor pressure p_0_(CO_2_) = 6.4121 MPa, p_0_(N_2_) = 97.152 kPa) [64,65].

MOF	Pore Fraction/% [56]	*ρ*/g cm^−3^	V_pore_ (cal.)/cm^3^ g^−1^	V_pore_ (CO_2_)/cm^3^ g^−1^	V_pore_ (N_2_)/cm^3^ g^−1^	S_BET_/m^2^ g^−1^
**1**	- ^a^	- ^a^	- ^a^	0.33	0.26	580
**2**	- ^a^	- ^a^	- ^a^	0.22	0.16	345
**3**	39	1.244	0.31	0.36	0.26	648
**4**	34	1.306	0.26	0.26	0.25	680
**5**	28	1.357	0.21	0.19	0.14	319

^a^ No value due to unavailable single crystal structure data.

**Table 5:** Cu content, as well as turnover frequency TOF in the conversion of cyclohexene with TBHP over **1**–**5** as catalysts after 7 h (T = 323 K, V_chloroform_ = 15 cm^3^, c_cyclohexene_ = 0.86 mol L^−1^, n_Cyhex_/n_TBHP_ = 1/2, n_Cu,cat_ = 0.46 mmol).

Catalyst	Cu Content ^a^/wt %	TOF/h^−1^
**1**	21.9	2.8
**2**	21.2	3.1
**3**	22.5	2.6
**4**	21.9	3.0
**5**	18.7	4.4
Cu_3_(BTC)_2_	29.6	3.0
Cu(NO_3_)_2_	28.7	3.9

^a^ Determined by optical emission spectrometry with inductively coupled plasma (ICP-OES).

**Table 6:** Cyclohexene conversion X_Cyhex_, selectivity for 1-(*tert*-butylperoxy)-2-cyclohexene S_Cyhex-TBP_ as the main reaction product over MOFs investigated for the selective oxidation of cyclohexene with TBHP.

Catalyst	t/h	X_Cyhex_/%	S_Cyhex-TBP_/%	TOF/h^−1^	Reference
**5**	7	56	77	4.4	this work
MFU-1 ^a^	11	25	64	3.8	[73]
[Co^II^(BPD)]·3DMF ^b^	12	62	83	3.7	[74]
[Cu_2_L_2_] ^c^	7	82	56	6.6	[50]

^a^ Reaction conditions: cyclohexene (16 mmol), TBHP (8 mmol), 1,2,4-trichlorobenzene (2 mmol; as internal standard), MFU-1 (0.095 mmol based on Co), no solvent, 343 K; ^b^ Reaction conditions: cyclohexene (4 mmol), TBHP (12 mmol), 1,2,4-trichlorobenzene (4 mmol; as the internal standard), CH_2_Cl_2_ (5 cm^3^), [Co^II^(BPD)]·3DMF (0.056 mmol based on Co), no solvent, 353 K; ^c^ Reaction conditions: cyclohexene (25 mmol), TBHP in decane (50 mmol), chlorobenzene (25 mmol; as internal standard), CHCl_3_ (15 mL), [Cu_2_L_2_] (L = bis(carboxyphenyl)-1,2,4-triazole, 0.46 mmol based on Cu), 323 K).

## Supplementary Materials

Supplementary materials can be accessed at www.mdpi.com/1996-1944/10/4/338/s1. Figure S1: X-ray powder diffraction (PXRD) patterns of **1** (top left), **2** (top right) and **3** (bottom) after reflux synthesis and after various post-synthetic treatments, as well as simulation from single crystal data (*λ*(Cu-*K_α_*_1_) = 154.060 pm); Figure S2: X-ray powder diffraction (PXRD) patterns (*λ*(Cu-K_α1_) = 154.060 pm) of **4** (left) and **5** (right) after solvothermal and reflux synthesis, as well as after various post-synthetic treatments and simulation from single crystal data; Figure S3: PXRD patterns of Cu_3_(BTC)_2_ after activation at 393 K in air (before the catalytic experiment) and after use in the catalytic conversion of cyclohexene with TBHP for 7 h (after the catalytic experiment; T = 323 K, V_chloroform_ = 15 cm^3^, c_cyclohexene_ = 0.86 mol L^−1^, n_CyO_/n_TBHP_ = 1/2, m_cat_ = 100 mg); Figure S4: Temperature-dependent X-ray powder diffraction (TD-PXRD) patterns of **1**–**5** after Soxhlet extraction with methanol (*λ*(Cu-K_α1_) = 154.060 pm); Figure S5: Simultaneous thermal analysis (TG-DTA-MS) of **1** (top) and **2** (bottom). MS-signals of (H_2_O)^+^ (*m*/*z* = 18) and (MeO)^+^ (*m*/*z* = 31), as well as (MeCN)^+^ (*m*/*z* = 41) and (CO_2_)^+^ (*m*/*z* = 44) illustrate the evaporation of guest molecules and the decomposition of the framework, respectively; Figure S6: Simultaneous thermal analysis (TG-DTA-MS) of **3** (top) and **4** (bottom). MS-signals of (H_2_O)^+^ (*m*/*z* = 18) and (MeO)^+^ (*m*/*z* = 31), as well as (MeCN)^+^ (*m*/*z* = 41) and (CO_2_)^+^ (*m*/*z* = 44) illustrate the evaporation of guest molecules and the decomposition of the framework, respectively; Figure S7: Simultaneous thermal analysis (TG-DTA-MS) of **5** (top) and **3** after activation under vacuum (bottom) at 313 K for 24 h. MS-signals of (H_2_O)^+^ (*m*/*z* = 18), (MeCN)^+^ (*m*/*z* = 41) and (CO_2_)^+^ (*m*/*z* = 44) illustrate the decomposition of the framework; Figure S8: Decomposition temperatures of **1**–**5** determined by simultaneous thermal analysis (TG-DTA-MS) and temperature-dependent powder X-ray diffraction (TD-PXRD); Figure S9: CO_2_ adsorption isotherms (298 K) of **1**–**5** (black symbols, first measurement; green symbols, second measurement); Figure S10: N_2_ adsorption isotherms (77 K, p_0_ = 97.152 kPa) of **1**–**5**. Closed symbols: adsorption, open symbols: desorption (lines are to guide the eyes); Figure S11: Conversion of cyclohexene X_Cyhex_ and TBHP X_TBHP_, as well as selectivity for 1-(*tert*-butylperoxy)-2-cyclohexene S_Cyhex-TBP_, cyclohexene oxide S_CyhexO_, 2-cyclohexen-1-one S_Cyhex-ene_, 2-cyclohexen-1-ol S_Cyhex-ol_ for compounds **1**–**5**, Cu_3_(BTC)_2_ (Basolite C300, Sigma-Aldrich) and the homogeneously dissolved Cu(NO_3_)_2_ (99.5%, Merck) after a reaction time of 7 h (T = 323 K, V_chloroform_ = 15 cm^3^, c_cyclohexene_ = 0.86 mol L^−1^, n_Cyhex_/n_TBHP_ = 1/2, n_Cu,cat_ = 0.46 mmol); Figure S12: Selectivity for 1-(*tert*-butylperoxy)-2-cyclohexene S_Cyhex-TBP_ for compounds **1**–**5** and Cu_3_(BTC)_2_ different catalysts as a function of cyclohexene conversion X_Cyhex_ during 1–7 h of reaction time at 323 K in liquid chloroform (reaction conditions as in Figure S11); Figure S13: Conversion of cyclohexene X_Cyhex_ as a function of reaction time over **5** (left) and over **3** (right) and after removal of the catalyst by hot filtration after 3 h of reaction (reaction conditions as in Figure S11). In both diagrams, the conversion in the absence of a catalyst is also included; Figure S14: Conversion of cyclohexene X_Cyhex_ and TBHP X_TBHP_, as well as selectivity for 1-(*tert*-butylperoxy)-2-cyclohexene S_Cyhex-TBP_, cyclohexene oxide S_CyhexO_, 2-cyclohexen-1-one S_Cyhex-ene_, 2-cyclohexen-1-ol S_Cyhex-ol_ over Cu-containing MOF catalysts after a reaction time of 5 h (reaction conditions as in Figure S11) before (run 1) and after separation and regeneration (run 2) of the catalyst (under vacuum at 323 K for 16 h for **5** and **3** and in air at 393 K for 16 h for Cu_3_(BTC)_2_); Figure S15: CO_2_ adsorption isotherms (298 K) of **3** and **5** before and after catalytic experiments (lines are to guide the eyes); Table S1: Synthesis method, ligand, metal salt, solvent, product and product yield for Compounds **1**–**5**; Table S2: Single crystal structure data of compounds **3**–**5**.
