# Supplementary material for: A Series of Robust Copper-Based Triazolyl Isophthalate MOFs: Impact of Linker Functionalization on Gas Sorption and Catalytic Activity
Source: Materials (Basel). 2017 Mar 24;10(4):338. doi: 10.3390/ma10040338 (PMC5506898; doi:10.3390/ma10040338)
Supplement: Supplementary file 1 [file materials-10-00338-s001.pdf]

"A series of robust copper-based triazolyl isophtalate MOFs: impact of linker functionalization on gas sorption and catalytic activity"

By *Ulrike Junghans, Merten Kobalz, Oliver Erhart, Hannes Preißler, Jörg Lincke, Jens Möllmer, Harald Krautscheid, Roger Gläser*

Electronic Supporting Information (ESI)

1. MOF syntheses

Method A: Solvothermal synthesis

A stainless steel autoclave (PARR) with polytetrafluoroethene insert was loaded with the ligand, metal salt and solvent (cf. Table S1) and sealed. The reaction mixture was heated within one hour to 413 K. The temperature was kept at a constant level for five hours, then the autoclave was cooled to room temperature during a period of 60 hours. The crystalline product was washed with the same solvent as used for the synthesis.

Method B: Reflux synthesis

Respective amounts of ligand and metal salt (cf. Table S1) were suspended in the solvent and heated under reflux for 48 h. Afterwards, the reaction mixture was cooled to room temperature, the solid product was filtered off and washed thoroughly with the same solvent as used for the synthesis.

Table S1. Synthesis method, ligand, metal salt, solvent, product and product yield for the compounds **1–5**.

compound	method	ligand	metal salt	solvent	product	yield
$^3_\infty$[Cu$_4$(μ_3-OH)$_2$(H-Me-trz-ia)$_3$] (1)	B	3.75 mmol H_2(H-Me-trz-ia)	5.0 mmol Cu(OAc)$_2$ · H_2O (98%, Sigma-Aldrich)	150 ml H_2O/MeOH (99,8%, AnalaR NORMAPUR® ACS, VWR)	blue microcrystalline powder	1.52 g
$^3_\infty$[Cu$_4$(μ_3-OH)$_2$(H-Et-trz-ia)$_3$] (2)	B	3.75 mmol H_2(H-Et-trz-ia)	5.0 mmol Cu(OAc)$_2$ · H_2O	150 ml H_2O/MeOH	blue microcrystalline powder	2.00 g
$^3_\infty$[Cu$_4$(μ_3-OH)$_2$(Me$_2$-trz-ia)$_3$(H_2O)] (3)	A	0.1 mmol H_2(Me$_2$-trz-ia)	0.1 mmol $CuSO_4$ · 5 H_2O (98%, Sigma-Aldrich)	5 ml H_2O/MeCN (99,9%, CHROMASOLV™, VWR)	blue crystals	few crystals
	B	3.75 mmol H_2(Me$_2$-trz-ia)	5.0 mmol Cu(OAc)$_2$ · H_2O	150 ml H_2O/MeOH	blue microcrystalline powder	1.57 g
$^3_\infty$[Cu$_4$(μ_3-OH)$_2$(Me-Et-trz-ia)$_3$(H_2O)$_2$] (4)	A	0.1 mmol H_2(Me-Et-trz-ia)	0.13 mmol Cu(OAc)$_2$ · H_2O	5 ml H_2O	blue crystals	34.90 mg
	B	5.0 mmol H_2(Me-Et-trz-ia)	6.67 mmol Cu(OAc)$_2$ · H_2O	100 ml H_2O	blue microcrystalline powder	2.57 g
$^3_\infty$[Cu$_4$(μ_3-OH)$_2$(Et$_2$-trz-ia)$_3$(H_2O)] (5)	A	0.1 mmol H_2(Et$_2$-trz-ia)	0.1 mmol Cu(OAc)$_2$ · H_2O	5 ml H_2O/MeOH	blue crystals	22.70 mg
	B	3.75 mmol H_2(Et$_2$-trz-ia)	5.0 mmol Cu(OAc)$_2$ · H_2O	150 ml H_2O/MeOH	blue microcrystalline powder	1.57 g

2. Characterization of the MOFs

2.1. Single crystal structure data

The X-ray diffraction data were obtained using an IPDS-2T image plate diffractometer with Mo-K_α radiation (λ = 71.073 pm). The data sets were processed with the program STOE X-AREA [1]. The structures were solved by direct methods and refined using SHELX-2014 [2]. The non-hydrogen atoms of the frameworks were refined anisotropically. The coordinates of the hydrogen atoms of the frameworks were calculated for idealized positions. Due to the fact that no solvent molecules could be localized in the difference Fourier map, the SQUEEZE-routine of the program PLATON was applied [3]. The program DIAMOND 3.2f was used to visualize the structures [4].

Table S2. Single crystal structure data of the compounds **3–5**.

compound	3	4	5
crystal color and shape	blue prism	blue prism	blue prism
crystal size / mm	0.24 · 0.25 · 0.39	0.36 · 0.43 · 0.48	0.24 · 0.27 · 0.30
formula	$C_{36}H_{31}N_9O_{15}Cu_4$	$C_{39}H_{39}N_9O_{16}Cu_4$	$C_{42}H_{43}N_9O_{15}Cu_4$
molar mass M / g mol^{-1}	1083.86	1143.95	1168.01
temperature / K	180(2)	180(2)	180(2)
diffractometer	STOE IPDS-2T	STOE IPDS-2T	STOE IPDS-2T
wavelength / pm	71.073	71.073	71.073
crystal system	orthorhombic	orthorhombic	orthorhombic
space group	*Pbca* (no. 61)	*Pbca* (no. 61)	*Pbca* (no. 61)
unit cell parameters / pm, °	a = 1875.00(8)<br>b = 2428.78(6)<br>c = 2540.78(7)	a = 1872.71(3)<br>b = 2461.21(5)<br>c = 2525.07(6)	a = 1854.69(5)<br>b = 2420.12(7)<br>c = 2547.32(7)
Volume V / 10^6 pm^3	11570.6(7)	11638.4(4)	11433.8(6)
formula units Z per unit cell	8	8	8
density / g cm^{-3}	1.244	1.306	1.357
absorption coefficient μ (Mo-Kα) / mm^{-1}	1.508	1.505	1.532
θ range / °	1.0–25.0	0.96–27.00	0.96–27.00
number of measured reflections	53354	73397	60723
number of independent reflections	10189	12698	12467
number of observed reflections (I>2σ(I))	8339	9109	9063
R_{int}	0.0681	0.0576	0.0505
parameters	595	605	648
R_1	0.0708*	0.0580*	0.0418*
wR_2	0.2035*	0.1715*	0.1063*
max. / min. residual e$^-$-density / 10^{-6} pm^{-3}	2.4 / -0.9	1.7 / -0.9	0.7 / -1.0

* The PLATON/SQUEEZE routine was used to remove the diffuse residual electron density as no solvent molecules could be localized during single crystal structure refinement.

2.2. *X-ray powder diffraction (PXRD) and thermal stability*

PXRD

The PXRD patterns were recorded on a STOE STADI-P diffractometer using Cu-K$_{\alpha 1}$ radiation (λ = 154.060 pm) [5]. The samples were prepared in glass capillaries (Hilgenberg, outer diameters 0.5 mm or 0.7 mm). The temperature-dependent PXRD experiments (TD-PXRD) were carried out on a STOE STADI-P diffractometer with a high-temperature attachment using Cu-K$_{\alpha 1}$ radiation (λ = 154.060 pm). Prior to each measurement, all samples were Soxhlet-extracted with methanol. After drying in air, the samples were filled into glass capillaries (Hilgenberg, outer diameter 0.5 mm). The PXRD data were taken in steps of 5 K from 303 K to 773 K.

Soxhlet extraction with methanol was carried out over seven days to achieve a complete solvent exchange. After this postsynthetic treatment, the PXRD patterns remain unchanged. Even after multiple adsorption-desorption cycles including activation procedures, the reflection positions match the pristine patterns. Merely a slight broadening of the reflections is observed after adsorption and catalytic experiments (Figure S1, Figure S2).

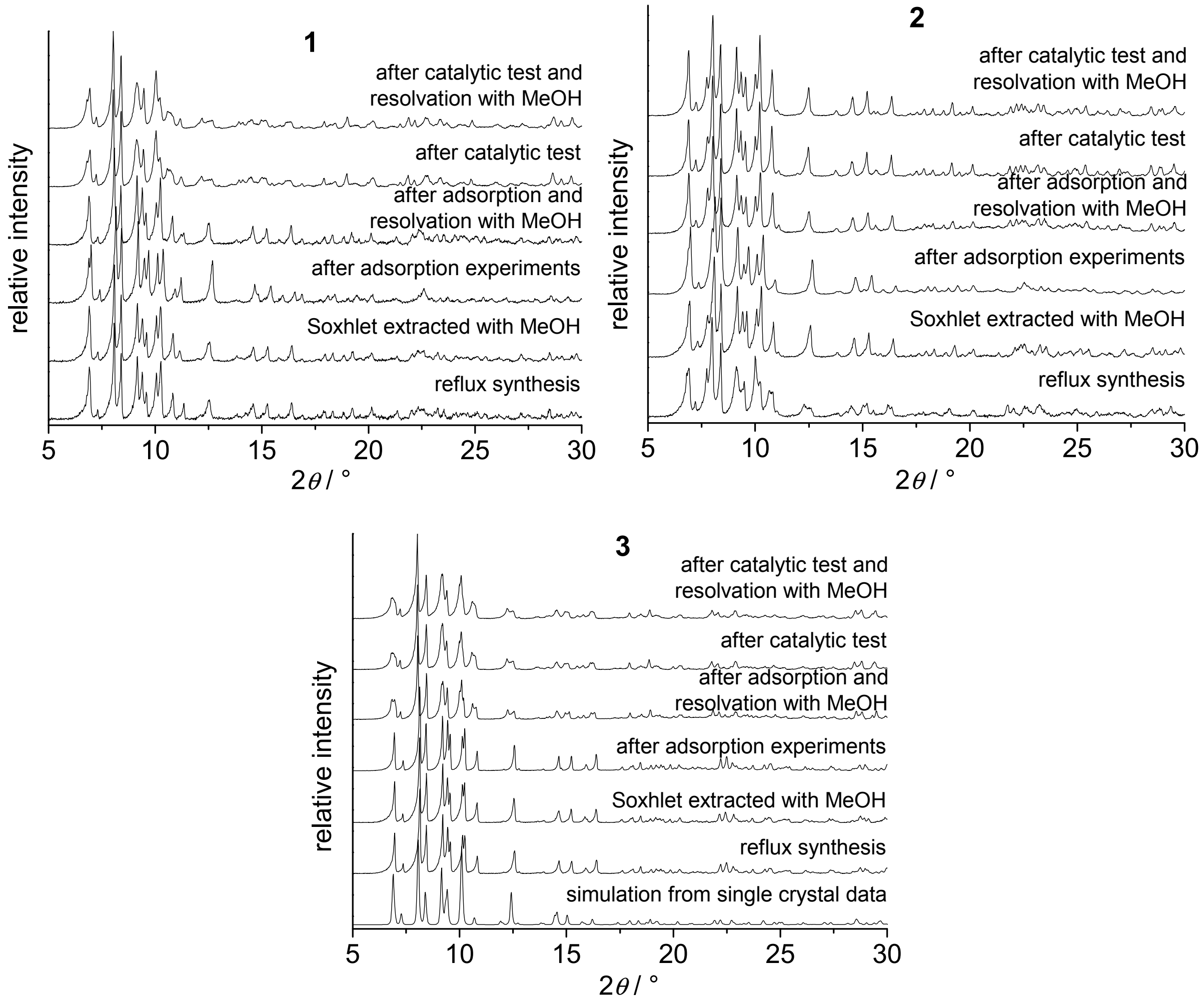

Figure S1. X-ray powder diffraction (PXRD) patterns of **1** (top left), **2** (top right) and **3** (bottom) after reflux synthesis and after various postsynthetic treatments as well as simulation from single crystal data (λ(Cu-$K_{\alpha 1}$) = 154.060 pm).

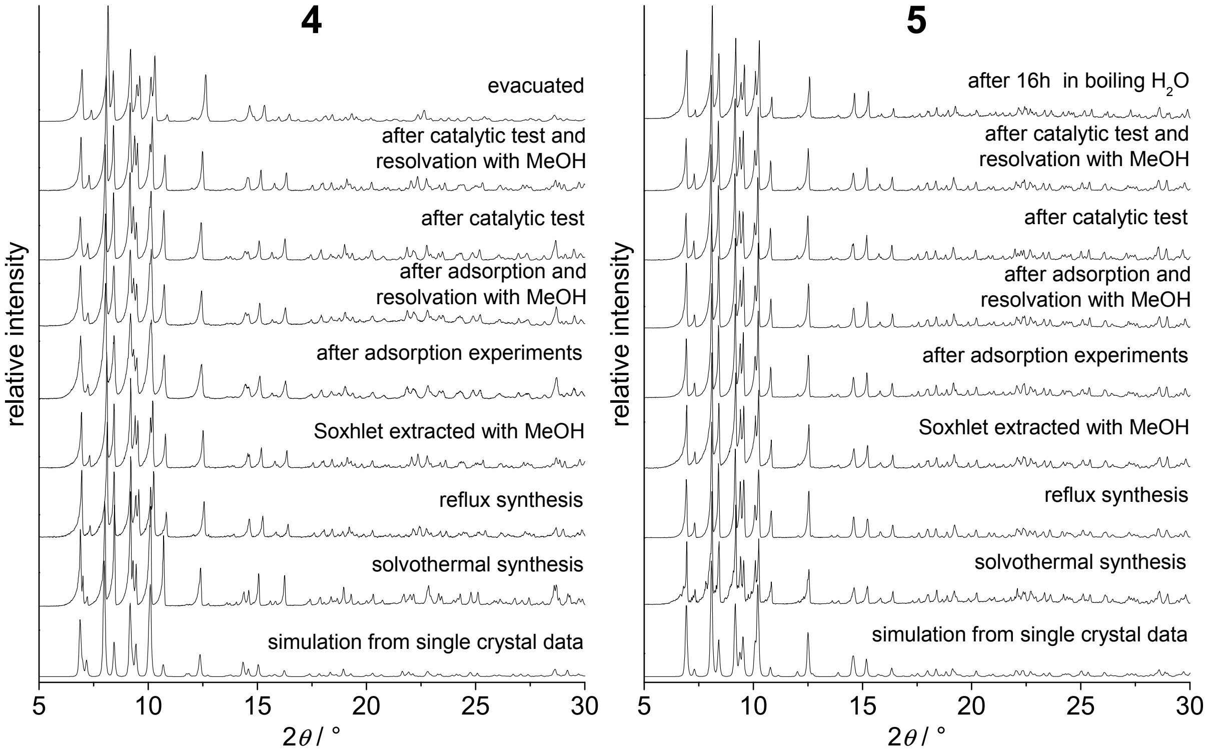

Figure S2. X-ray powder diffraction (PXRD) patterns (λ(Cu-K$_{\alpha1}$) = 154.060 pm) of **4** (left) and **5** (right) after solvothermal and reflux synthesis as well as after various postsynthetic treatments and simulation from single crystal data.

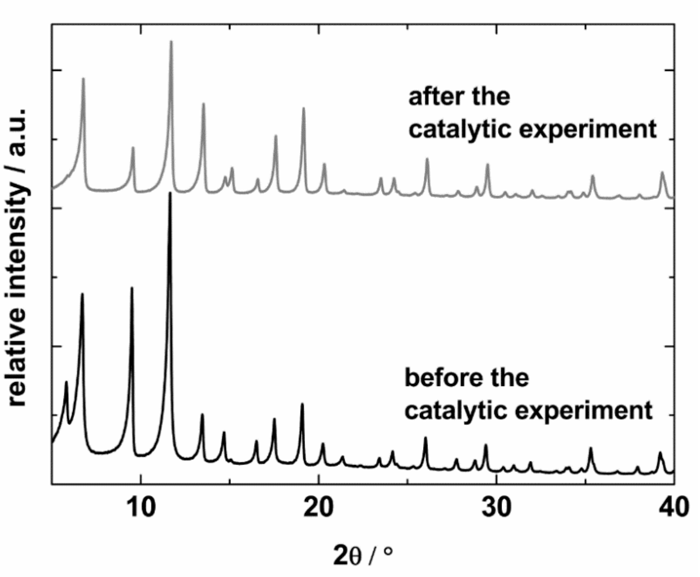

Figure S3. PXRD patterns of $Cu_3(BTC)_2$ after activation at 393 K in air (before the catalytic experiment) and after use in the catalytic conversion of cyclohexene with TBHP for 7 h (after the catalytic experiment; T = 323 K, $V_{chloroform}$ = 15 cm^3, $c_{cyclohexene}$ = 0.86 mol L^{-1}, n_{CyO}/n_{TBHP} = 1/2, m_{cat} = 100 mg).

TD-PXRD

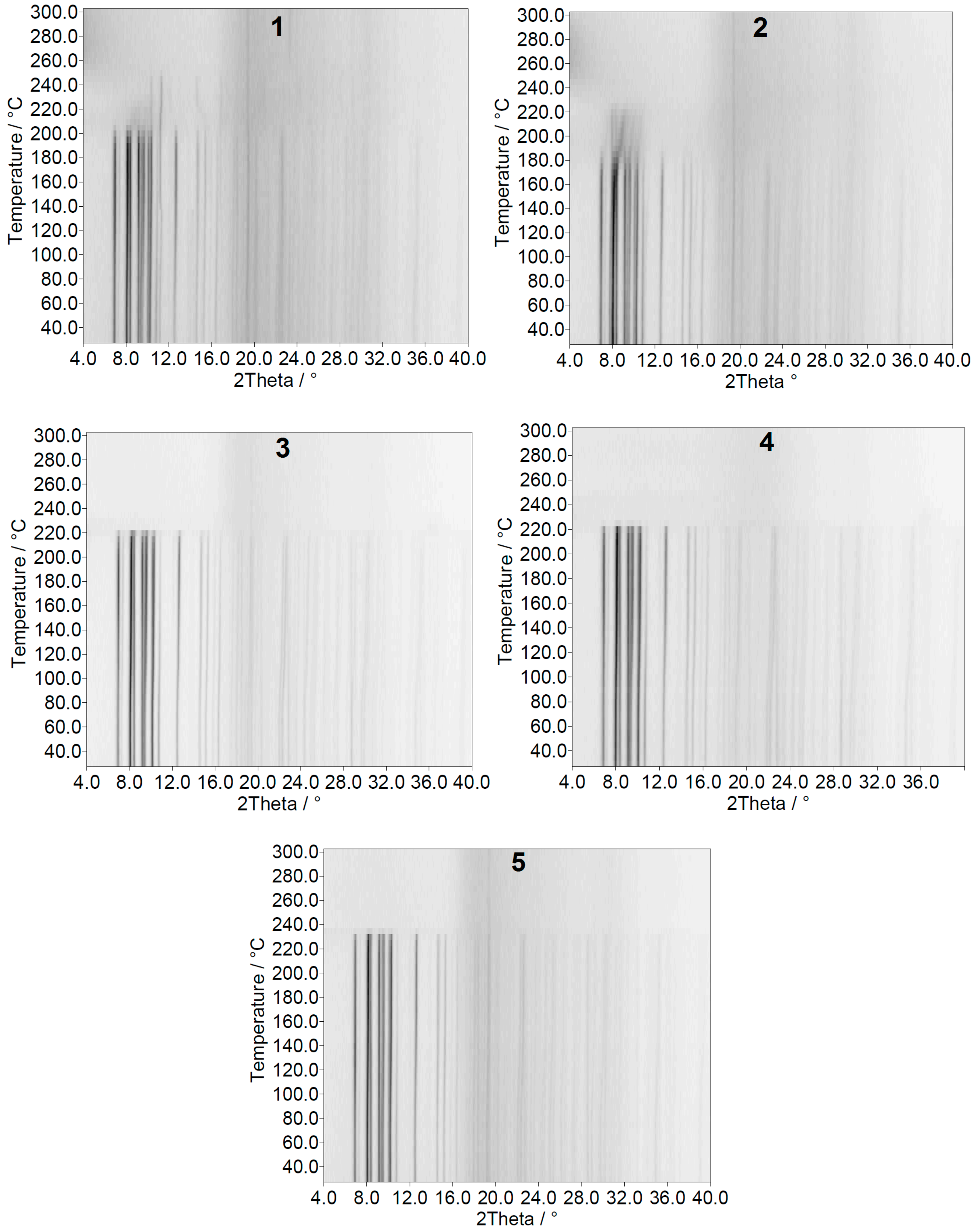

Figure S4. Temperature-dependent X-ray powder diffraction (TD-PXRD) patterns of **1**–**5** after Soxhlet extraction with methanol (λ(Cu-K$_{\alpha1}$) = 154.060 pm).

TG-DTA-MS

The simultaneous thermal analyses (TG-DTA-MS) were carried out using corundum crucibles on a STA 449 *F1* Jupiter (Netzsch) coupled to an Aeolos QMS 403C mass spectrometer. The samples were heated at a rate of 10 K min^{-1} up to 873 K under constant flow (50 mL min^{-1}) of Helium (99.999%, Air Products). All samples were Soxhlet-extracted with methanol (99,8%, AnalaR NORMAPUR® ACS, VWR). Prior to each analysis, the sample was evacuated for 5 min at room temperature in the instrument.

The individual diagrams of the simultaneous thermal analyses of **1–5** after Soxhlet extraction with methanol are shown in Figures S5-S7. All materials show a mass loss up to 200 °C, which is related to the evaporation of guest molecules. The amount of solvent leaving the pore system varies within the series. Note, that a short pre-measurement evacuation procedure at room temperature inside the instrument was applied for all samples. After the pretreatment, the networks contain no or only small amounts of methanol. Instead, the evaporation of water is detected at elevated temperatures. It can be concluded that the postsynthetic solvent exchange by Soxhlet extraction with methanol is not complete. This is probably due to the narrow pore system of the MOFs, which is not completely accessible for methanol molecules. Additionally, a large portion of the embedded methanol is already evaporated during evacuation before the analysis. The multi-step water loss results from different degrees of adsorptive interaction with pores of various diameters. Hence, the desorption of the water molecules is observed in different temperature ranges. The last peak in the MS-signal of water (m/z = 18 $(H_2O)^+$) is assigned to the removal of coordinated water molecules and bridging hydroxide ions. This removal is accompanied by the thermal decomposition of the frameworks, associated with the release of CO_2 from the organic linker. In case of the methyl- and dimethyl-substituted networks (**1** and **3**), the characteristic fragment $(NCCH_3)^+$ is detected (m/z = 41). Note that the simultaneous thermal analysis of **3** after activation at 313 K for 24 h shows almost no mass loss up to the decomposition temperature (Figure S7). Hence, after this activation procedure, no guest molecules are present in the pore system of the framework.

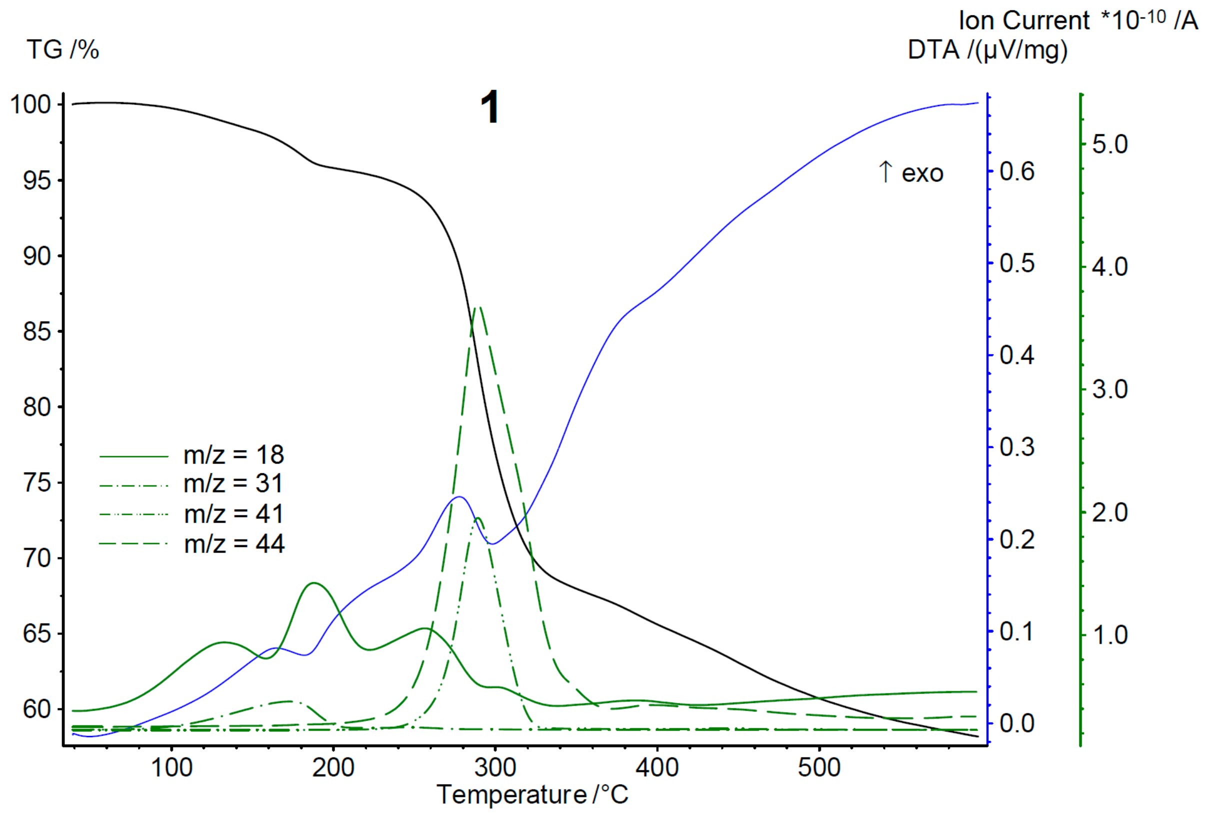

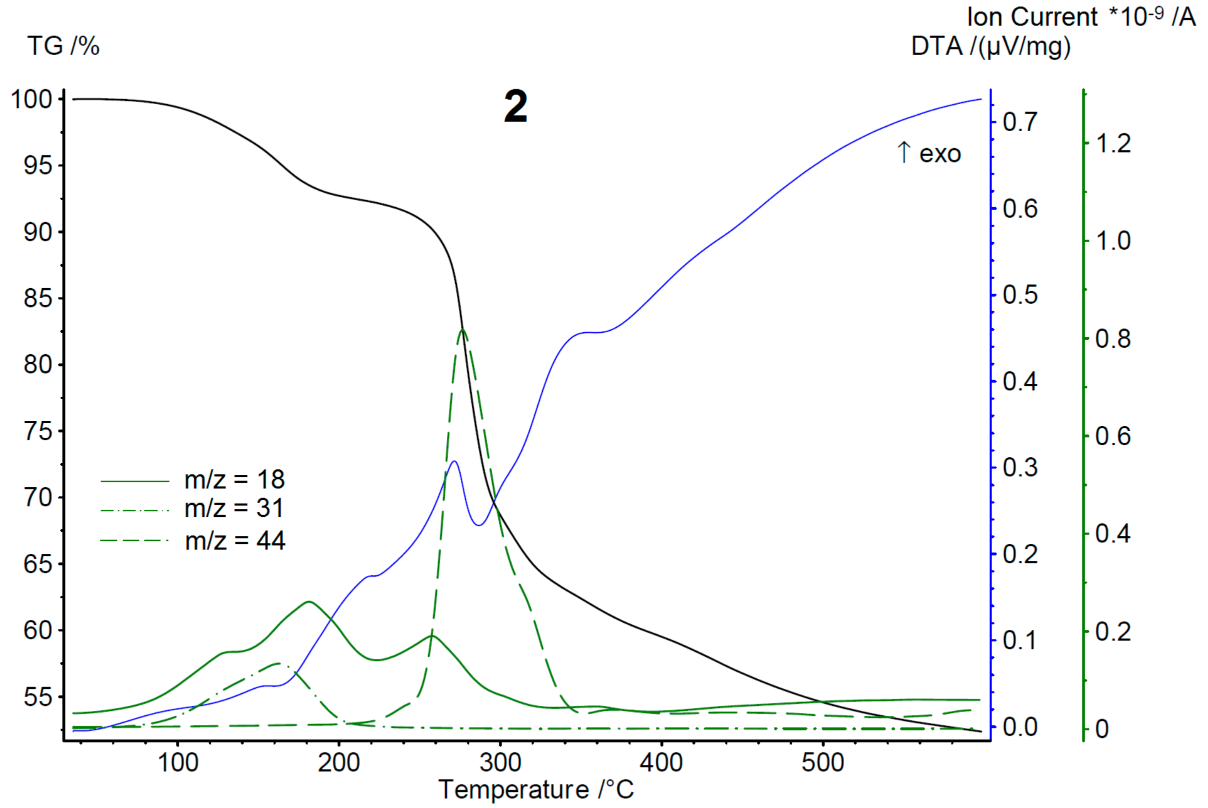

Figure S5. Simultaneous thermal analysis (TG-DTA-MS) of **1** (top) and **2** (bottom). MS-Signals of $(H_2O)^+$ (m/z = 18) and $(MeO)^+$ (m/z=31) as well as $(MeCN)^+$ (m/z = 41) and $(CO_2)^+$ (m/z = 44) illustrate the evaporation of guest molecules and decomposition of the framework, respectively.

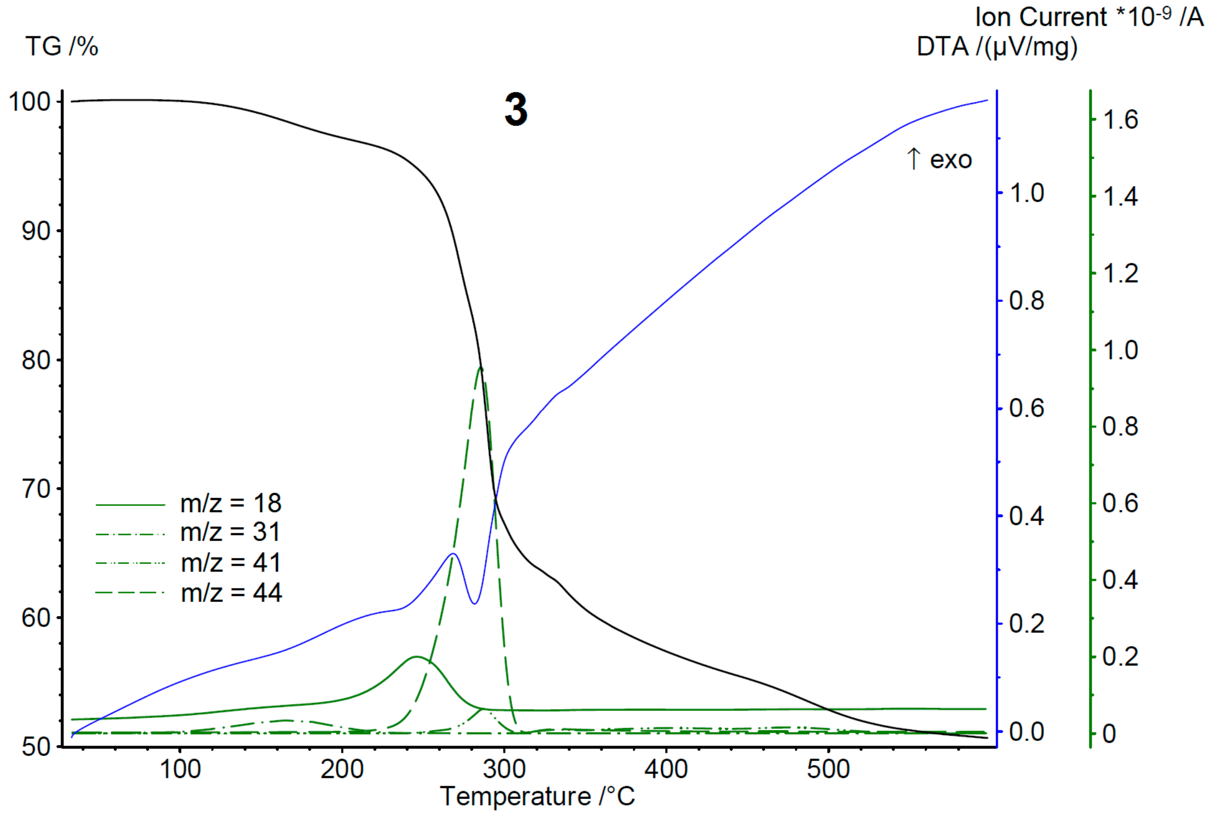

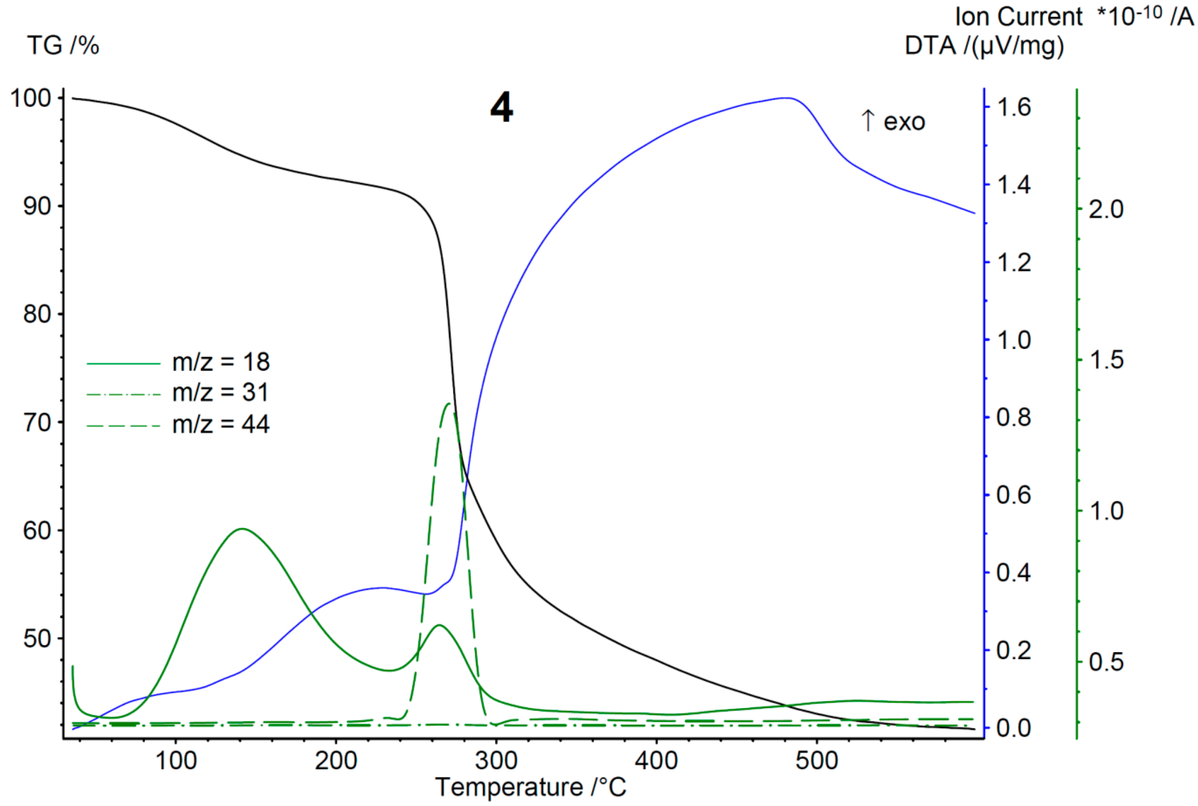

Figure S6. Simultaneous thermal analysis (TG-DTA-MS) of **3** (top) and **4** (bottom). MS-Signals of $(H_2O)^+$ (m/z = 18) and $(MeO)^+$ (m/z = 31) as well as $(MeCN)^+$ (m/z = 41) and $(CO_2)^+$ (m/z = 44) illustrate the evaporation of guest molecules and decomposition of the framework, respectively.

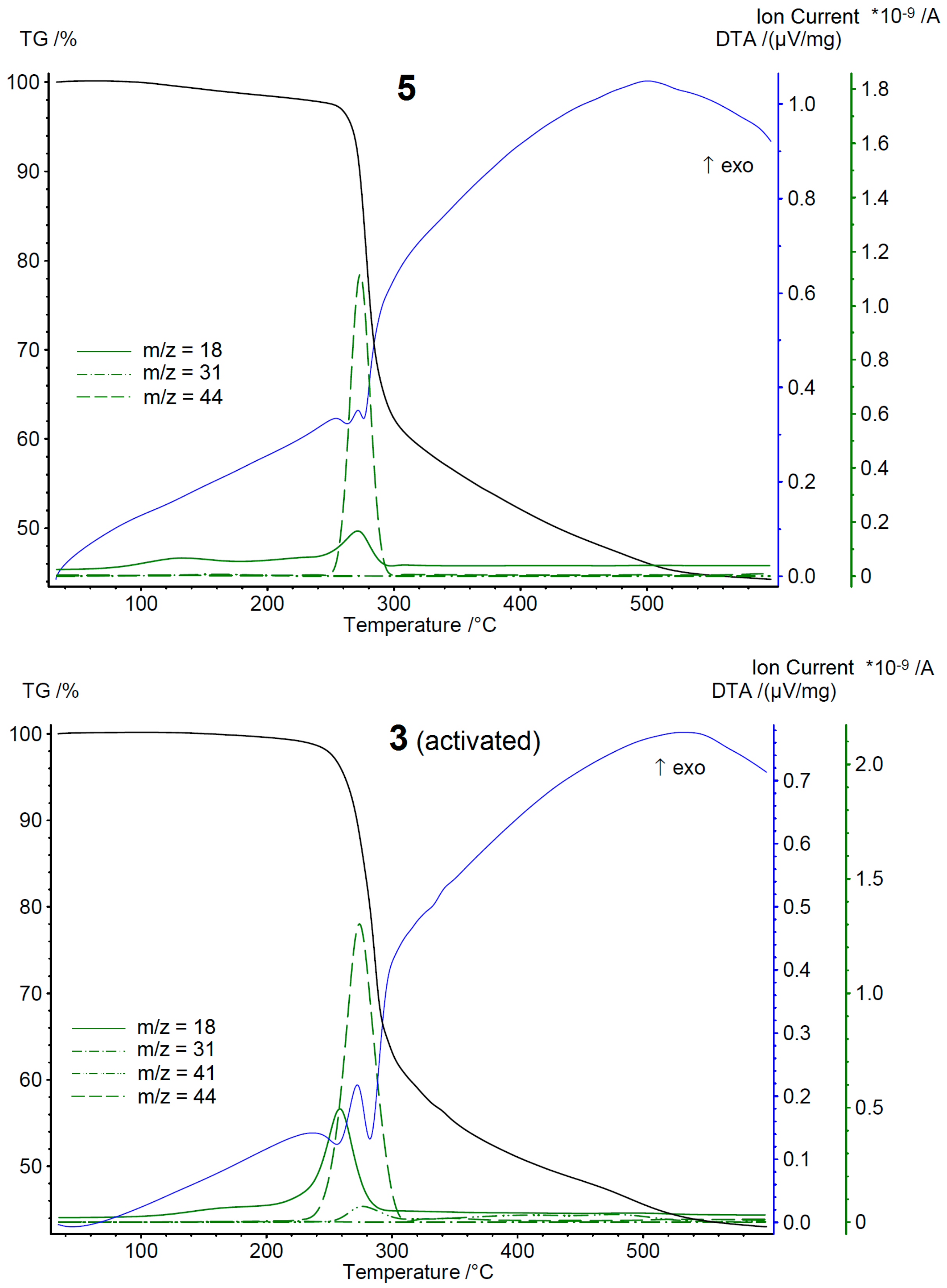

Figure S7. Simultaneous thermal analysis (TG-DTA-MS) of **5** (top) and **3** after activation under vacuum (bottom) at 313 K for 24 h. MS-Signals of $(H_2O)^+$ (m/z = 18), $(MeCN)^+$ (m/z = 41) and $(CO_2)^+$ (m/z = 44) illustrate the decomposition of the framework.

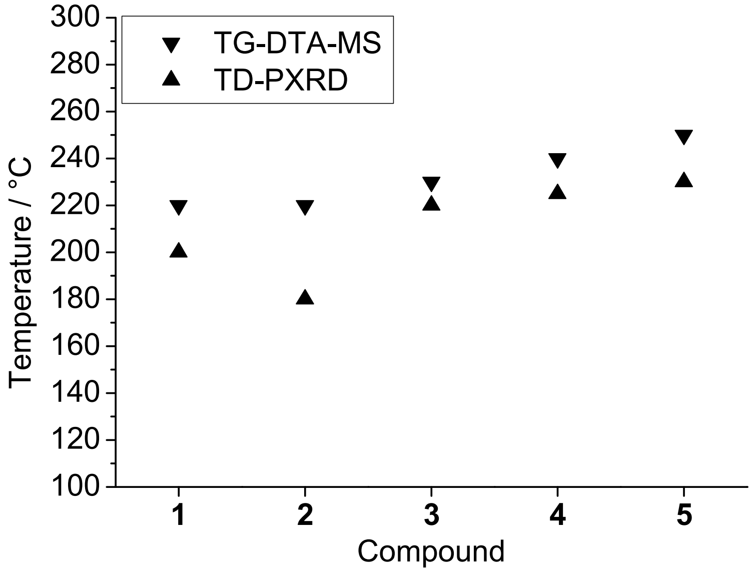

Figure S8. Decomposition temperatures of **1–5** determined by simultaneous thermal analysis (TG-DTA-MS) and temperature dependent powder X-ray diffraction (TD-PXRD).

2.3. Adsorption studies

Materials

All gases were obtained from Air Products (USA) with purities of 99.995% for CO_2, 99.995% for N_2 and 99.9992% for He, respectively). The microcrystalline MOF samples were treated by Soxhlet extraction over seven days in order to remove synthesis residues blocking the pore system. For adsorption studies, ca. 0.3 g sample were transferred into the glass sample cell for experiments with N_2 or a stainless steel sample holder for experiments with CO_2, respectively. Prior to each adsorption experiment, the samples were evacuated at 313 K for 24 h under vacuum of a turbomolecular pump. N_2 adsorption experiments at 77 K were performed using the commercially available automatic manometric system BELSORP-max (BEL, Japan).

High pressure adsorption of CO_2 was carried out in a magnetic suspension balance (Rubotherm, Germany). Various pressure transducers (Newport Omega, US) in a range from vacuum up to 16 MPa with an accuracy of 0.05% were applied. The gas was dosed into the sample chamber at elevated pressures. Equilibrium was assumed when no further weight increase was detected within 15 min. The temperature was kept constant with an accuracy of ± 0.5 K in each experiment. Additionally, for each isotherm, a buoyancy correction was used to calculate the surface excess mass from the measured values. A detailed description of this procedure can be found in the literature [6]. For the determination of the fluid density, the program *FLUIDCAL* was used for each gas [7–9].

CO_2 adsorption isotherms

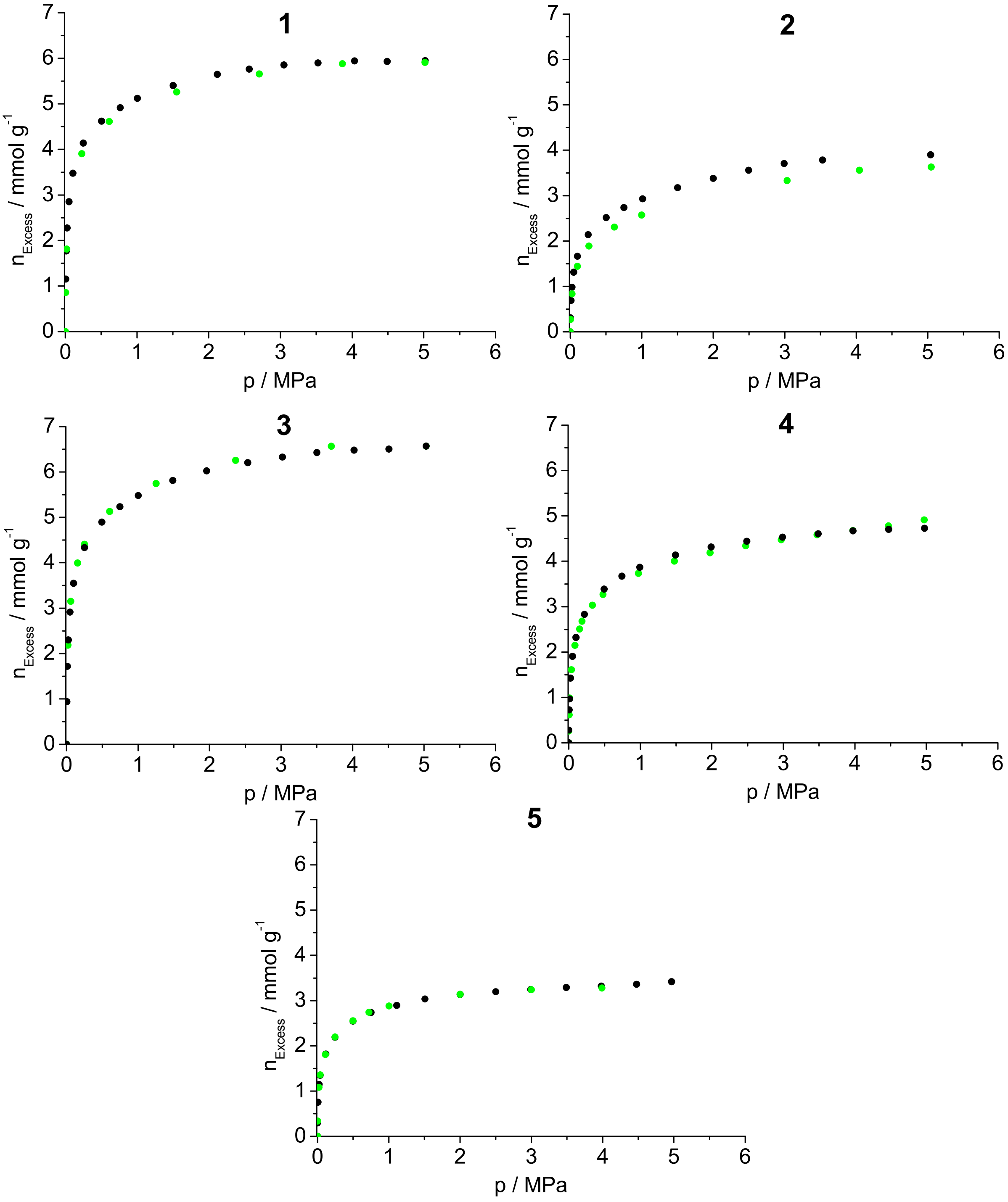

Figure S9. CO_2 adsorption isotherms (298 K) of **1–5** (black symbols first measurement, green symbols second measurement).

N_2 adsorption isotherms

Though N_2 is adsorbed by all materials, **1**, **3** and **4** show approximately the same N_2 uptake (V_{STP} ~ 150 cm^3 g^{-1}). However, the calculated pore volumes differ significantly. Merely in case of **4,** the experimentally determined pore volume is in good agreement with the calculated value based on crystal structure data (cf. Table 4 in the manuscript). For the other MOFs, the experimentally determined specific pore volumes (N_2) differ significantly from the calculated values and also from the values determined by CO_2 adsorption.

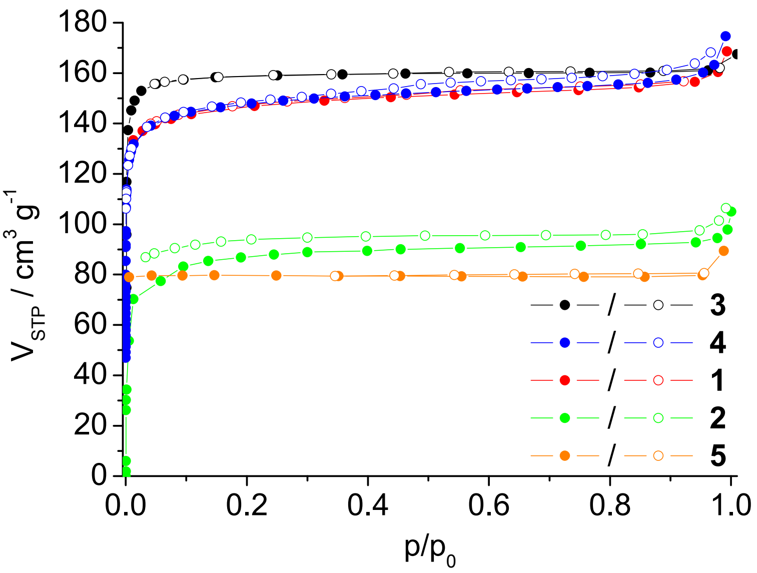

Figure S10. N_2 adsorption isotherms (77 K, p_0 = 97.152 kPa) of **1–5**. Closed symbols: adsorption, open symbols: desorption (lines are to guide the eyes).

3. Catalytic selective oxidation of cyclohexene with *tert*-butylhydroperoxide (TBHP)

3.1. Catalytic activity of 1–5 and of reference catalysts

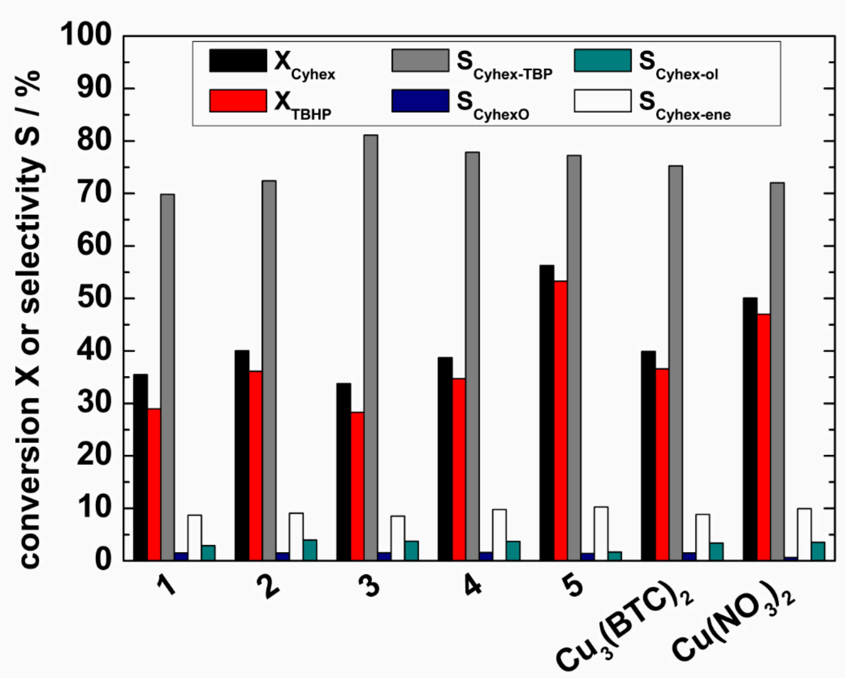

Figure S11. Conversion of cyclohexene X_{Cyhex} and TBHP X_{TBHP} as well as selectivity for 1-(*tert*-butylperoxy)-2-cyclohexene $S_{Cyhex\text{-}TBP}$, cyclohexene oxide S_{CyhexO}, 2-cyclohexen-1-one $S_{Cyhex\text{-}ene}$, 2-cyclohexen-1-ol $S_{Cyhex\text{-}ol}$ for the compounds **1-5**, $Cu_3(BTC)_2$ (Basolite C300, Sigma-Aldrich) and the homogeneously dissolved $Cu(NO_3)_2$ (99.5%, Merck) after a reaction time of 7 h (T = 323 K, $V_{chloroform}$ = 15 cm^3, $c_{cyclohexene}$ = 0.86 mol L^{-1}, n_{Cyhex}/n_{TBHP} = 1/2, $n_{Cu,cat}$ = 0.46 mmol).

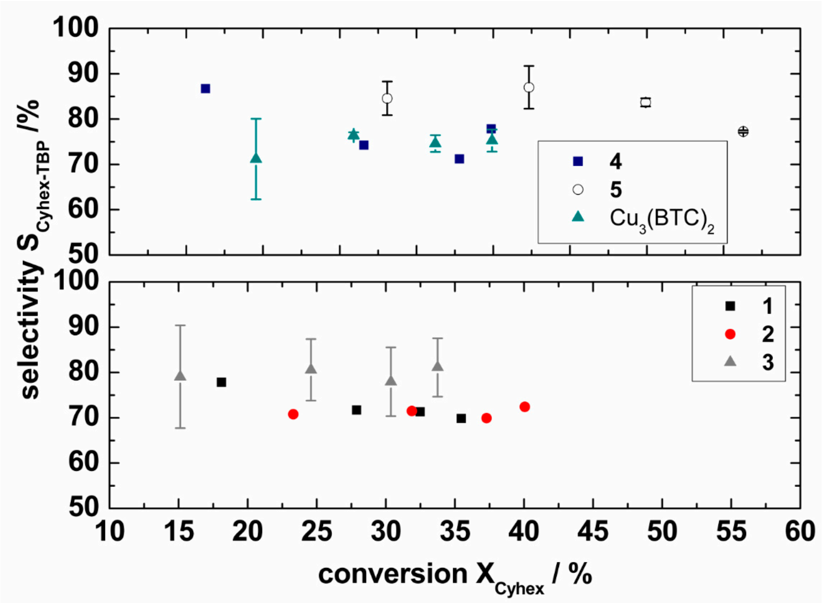

Figure S12. Selectivity for 1-(*tert*-butylperoxy)-2-cyclohexene $S_{Cyhex\text{-}TBP}$ for the compounds **1-5** and $Cu_3(BTC)_2$ different catalysts as a function of cyclohexene conversion X_{Cyhex} during 1–7 h of reaction time at 323 K in liquid chloroform (reaction conditions as in Figure S11).

3.2 Hot filtration experiments

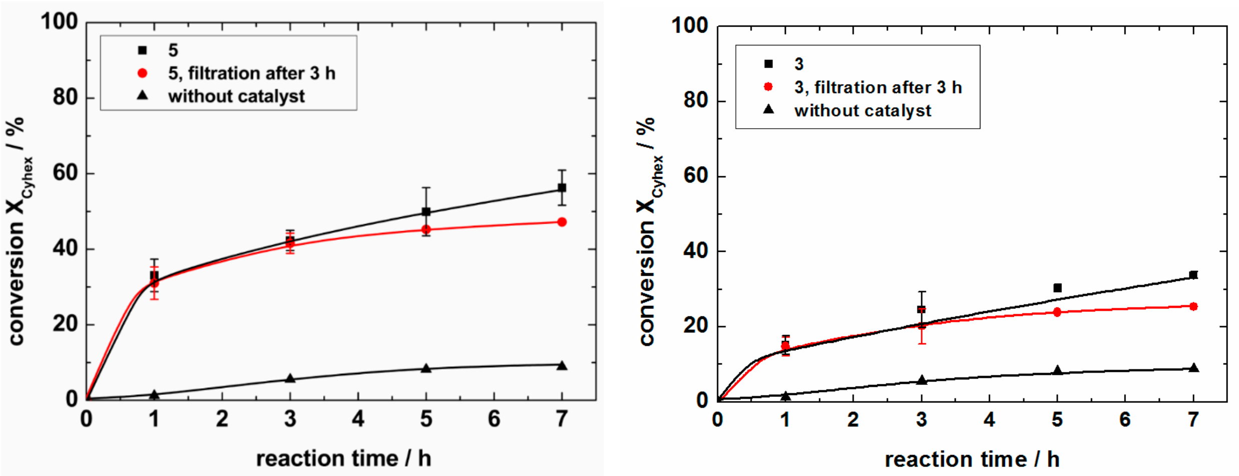

Figure S13. Conversion of cyclohexene X_{Cyhex} as a function of reaction time over **5** (left) and over **3** (right) and after removal of the catalyst by hot filtration after 3 h of reaction (reaction conditions as in Figure S11). In both diagrams the conversion in the absence of a catalyst is also included.

ICP-OES analysis

The metal content of **1–5** and $Cu_3(BTC)_2$ was determined by elemental analysis via optical emission spectrometry with inductively coupled plasma (ICP-OES, Optima 8000, Perkin Elmer) after dissolving 20 mg of the sample in a mixture of 2 cm^3 HF (48%, Sigma-Aldrich), 2 cm^3 of HNO_3 (69%, Sigma-Aldrich) and 2 cm^3 of HCl (35%, ROTIPURAN® Supra) by chemical extraction under autogenous pressure at 900 W and a rate of 10 W min^{-1} for 99 min in a microwave oven (Multiwave 3000; Anton Paar).

3.3 Reusability

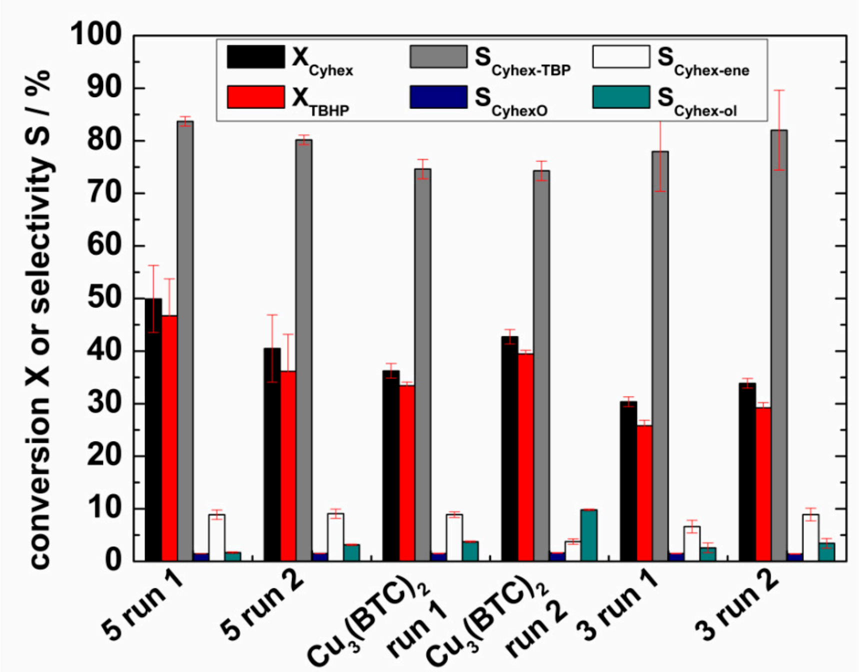

Figure S14. Conversion of cyclohexene X_{Cyhex} and TBHP X_{TBHP} as well as selectivity for 1-(*tert*-butylperoxy)-2-cyclohexene $S_{Cyhex-TBP}$, cyclohexene oxide S_{CyhexO}, 2-cyclohexen-1-one $S_{Cyhex-ene}$, 2-cyclohexen-1-ol $S_{Cyhex-ol}$ over Cu-containing MOF catalysts after a reaction time of 5 h (reaction conditions as in Figure S11) before (run 1) and after separation and regeneration (run 2) of the catalyst (under vacuum at 323 K for 16 h for **5** and **3** and in air at 393 K for 16 h for $Cu_3(BTC)_2$).

3.4 CO_2 adsorption measurements after catalytic experiments

Although, in case of **3**, a slightly lower CO_2 uptake is observed after the catalytic experiment, the specific pore volume still matches the value calculated based on crystal structure data (0.31 $cm^3\ g^{-1}$). In contrast, for **5**, a slightly higher CO_2 uptake is observed after the catalytic experiment compared to the pristine material with an experimentally determined pore volume of 0.21 $cm^3\ g^{-1}$, which is in good agreement with the calculated value (0.21 $cm^3\ g^{-1}$). It is most likely, that the complete pore system is accessible for CO_2 only after multiple activation procedures (activation conditions prior to each adsorption measurement: sample evacuation at 313 K for 24 h in vacuum).

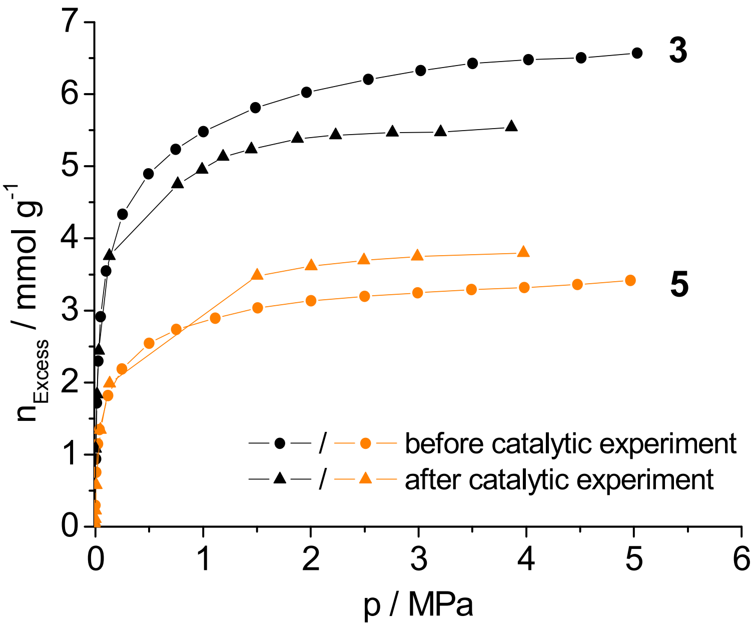

Figure S15. CO_2 adsorption isotherms (298 K) of **3** and **5** before and after catalytic experiments (reaction conditions as in Figure S11, lines are to guide the eyes).

References

1. *X-Area*; Stoe & Cie GmbH: Darmstadt, 2006.
2. Sheldrick, G.M. A short history of SHELX. *Acta Crystallogr., Sect. A: Found. Crystallogr.* **2008**, *64*, 112–122.
3. Spek, A.L. Single-crystal structure validation with the program PLATON. *J Appl Crystallogr* **2003**, *36*, 7–13.
4. Brandenburg. K: Diamond 3.2f Crystal Impact GbR, Bonn, 2010.
5. *WinXPow 3.0.2.7*; Stoe & Cie GmbH: Darmstadt, 2012.
6. Keller, J.; Staudt, R. *Gas Adsorption Equilibria*: *Experimental Methods and Adsorption Isotherms*; Springer: New York, 2004.
7. Span, R.; Wagner, W. A new equation of state for carbon dioxide covering the fuid region from the triple-point temperature to 1100 K at pressures up to 800MPa. *J. Phys. Chem. Ref. Data* **1996**, *25*, 1509–1596.
8. McCarty, R.D.; Arp, V.D. A new wide range equation of state for helium. *Adv. Cryog. Eng.* **1990**, *35*, 1465–1475.
9. Wagner, W. *FLUIDICAL*: Bochum, http://www.thermo.ruhr-uni-bochum.de/de/prof-w-wagner/software/fluidcal.htm, (status: 08.12.16).
